# Axonal and Dendritic Morphology of Excitatory Neurons in Layer 2/3 Mouse Barrel Cortex Imaged Through Whole-Brain Two-Photon Tomography and Registered to a Digital Brain Atlas

**DOI:** 10.3389/fnana.2021.791015

**Published:** 2022-01-25

**Authors:** Yanqi Liu, Georgios Foustoukos, Sylvain Crochet, Carl C.H. Petersen

**Affiliations:** Laboratory of Sensory Processing, Brain Mind Institute, Faculty of Life Sciences, École Polytechnique Fédérale de Lausanne (EPFL), Lausanne, Switzerland

**Keywords:** barrel cortex, axonal morphology, layer 2/3, two-photon tomography, mouse brain atlas

## Abstract

Communication between cortical areas contributes importantly to sensory perception and cognition. On the millisecond time scale, information is signaled from one brain area to another by action potentials propagating across long-range axonal arborizations. Here, we develop and test methodology for imaging and annotating the brain-wide axonal arborizations of individual excitatory layer 2/3 neurons in mouse barrel cortex through single-cell electroporation and two-photon serial section tomography followed by registration to a digital brain atlas. Each neuron had an extensive local axon within the barrel cortex. In addition, individual neurons innervated subsets of secondary somatosensory cortex; primary somatosensory cortex for upper limb, trunk, and lower limb; primary and secondary motor cortex; visual and auditory cortical regions; dorsolateral striatum; and various fiber bundles. In the future, it will be important to assess if the diversity of axonal projections across individual layer 2/3 mouse barrel cortex neurons is accompanied by functional differences in their activity patterns.

## Introduction

On the millisecond timescale, neurons communicate primarily by releasing neurotransmitters from presynaptic specializations along their axons in response to action potential firing, with the increased concentration of neurotransmitters acting to open ligand-gated ion channels largely concentrated in postsynaptic specializations on dendrites. In order for any two neurons to be synaptically connected it is essential that the axon of the presynaptic neuron is in close apposition to the dendrite of the postsynaptic neuron. Reconstructing the axon and dendrites of individual neurons, therefore, provides important information about where they might send and receive signals (Cajal, 1995). Whereas dendrites have relatively large diameters and are typically confined to a small region near the cell body of the neuron, axons have smaller diameters and can project long distances across the brain, spinal cord, and other parts of the body posing important challenges for their accurate characterization. Here, building on technical advances in single-cell anatomy developed in previous studies (Yamashita et al., 2013, 2018; Economo et al., 2016; Han et al., 2018; Winnubst et al., 2019; Peng et al., 2021), we further test procedures for whole-brain imaging, reconstruction, registration and quantification of the axonal and dendritic structure of single labeled neurons in layer 2/3 of the mouse barrel cortex.

The barrel cortex is a highly-specialized brain area serving as the primary whisker somatosensory cortex (wS1, also labeled SSp-bfd) for processing sensory information from the array of mystacial vibrissae (Brecht, 2007; Diamond et al., 2008; Petersen, 2019; Staiger and Petersen, 2021). Each whisker on the snout is mapped onto an anatomically-identifiable structure in layer 4 of wS1, known as a barrel, arranged somatotopically across the horizontal extent of wS1 (Woolsey and Van der Loos, 1970), helping with precise structure-function analyses. Bulk anterograde labeling of long-range axonal projections of neurons with cell bodies located in barrel cortex has revealed that they project to a large number of cortical and subcortical brain areas including secondary whisker somatosensory cortex (wS2, a part of SSs), whisker motor cortex (wM1/2, parts of MOp and MOs), perirhinal cortex, orbitofrontal cortex, secondary visual cortex, posterior parietal cortex, satellite cortical regions around wS1 including the dysgranular zone, contralateral cortex, different thalamic nuclei (VPM, higher-order posterior medial nucleus, and thalamic reticular nucleus), zona incerta, dorsolateral striatum, superior colliculus, anterior pretectal nucleus, pons, hypothalamus, and trigeminal nuclei (White and DeAmicis, 1977; Welker et al., 1988; Aronoff et al., 2010; Matyas et al., 2010; Mao et al., 2011; Sreenivasan et al., 2015; Guo et al., 2017; Sumser et al., 2017; Yamashita et al., 2018). Individual neurons appear to largely innervate only subsets of these targets, but the full extent of the anatomical diversity of long-range projection neurons in mouse wS1 is currently unknown. The local axonal arborizations within the barrel field of excitatory neurons with somata in wS1 has been characterized extensively (Schubert et al., 2001, 2006; Feldmeyer et al., 2006; Frick et al., 2008; Oberlaender et al., 2011; Feldmeyer, 2012; Narayanan et al., 2015; Staiger et al., 2015; Rojas-Piloni et al., 2017; Egger et al., 2020). A previous study reported the long-range axonal projections of infragranular pyramidal neurons, finding diverse corticofugal innervation patterns (Guo et al., 2017). Furthermore, sparse brain-wide labeling and imaging of genetically-defined neuronal populations revealed the morphology of various neurons in the primary somatosensory cortex, including some in the barrel field (Peng et al., 2021). Previous single-cell reconstruction studies targeted specifically to excitatory projection neurons in layer 2/3 of mouse wS1 have differentiated between two selected subsets depending upon retrograde labeling from wS2 and wM1 (Yamashita et al., 2013, 2018), allowing correlation with functional studies, which indicated interesting projection-specific differences in sensorimotor processing (Chen et al., 2013, 2015; Yamashita et al., 2013; Kwon et al., 2016; Yamashita and Petersen, 2016; Vavladeli et al., 2020). Here, in this study, we sampled layer 2/3 mouse barrel cortex neurons without pre-labeling of their long-range projections, and we made three important methodological advances over our previous work towards quantitatively studying their anatomy: (i) we imaged entire mouse brains using two-photon serial section tomography; (ii) we registered our data to a standardized digital atlas of the mouse brain; and (iii) we quantified axonal length in the context of brain areas annotated in the digital atlas. Through these technical advances, we have begun to further characterize the diversity of the axonal projections of individual layer 2/3 neurons in the mouse barrel cortex, adding to the important body of previous knowledge about the single-cell anatomy of excitatory projection neurons in the superficial layers of rodent primary whisker somatosensory cortex (Feldmeyer et al., 2006; Lübke and Feldmeyer, 2007; Yamashita et al., 2018; Egger et al., 2020; Peng et al., 2021; Staiger and Petersen, 2021). However, immunohistochemical labeling of processed tissue also pointed to important technical limitations indicating that our methodology revealed incomplete axonal arborizations.

## Materials and Methods

All animal procedures were performed in accordance with protocols approved by the Swiss Federal Veterinary Office (license VD1889.4).

### Head-Post Implantation

The experiments were carried out in 6–9 week-old male and female wild type C57BL/6J mice. Surgeries were performed under isoflurane anesthesia (4% for induction, then 1.5%) and the temperature was continuously monitored and held at 37°C using a closed-loop heating system (FHC Inc). All of the right whiskers, except the C2 whisker, were trimmed, and then mice were positioned in a stereotaxic frame using a nose clamp. In order to protect their eyes from drying, a hydrating eye gel was placed over the eyes during the surgery (VITA-POS, Pharma Medica AG). Pre-operative analgesia included intraperitoneal (i.p.) injections of Carprofen (0.3 ml at 0.5 mg/ml; Rimadyl, Pfizer) and subcutaneous injections of a mix of lidocaine (2% diluted 1:10) and bupivacaine (0.5% diluted 1:2) at the incision site. For postoperative analgesia, ibuprofen was administered through the water in the home cage drinking bottle for 4 days after surgery (2.5 ml in 250 ml of water bottle; Algifor Dolo Junior, VERFORA SA). In order to disinfect the skin before the incision, a povidone-iodine solution (Betadine, Mundipharma Medical Company) was used. Then a part of the scalp was removed using surgical scissors, the skull was exposed and the membrane of the periosteum was gently removed using a scalpel blade. The skull was then again disinfected, rinsed with Ringer solution, and subsequently fully dried using cotton buds. Afterward, a thin layer of cyanoacrylate glue was applied on the skull surface (Loctite 401, Henkel) and the metal head-post was placed on the right hemisphere. Finally, in order to strengthen the adhesion of the post to the skull, as well as to create a chamber for later procedures, dental cement (Paladur, Kulzer) was added. Immediately after the implantation surgery, the center of the barrel field was determined using intrinsic optical signal (IOS) imaging, as previously described (Ferezou et al., 2007; Le Merre et al., 2018; Yamashita et al., 2018). At the end of this procedure, the exposed skull was protected using a silicone elastomer (Kwik-Cast, WPI).

### Single-Cell Electroporation

After full recovery from the implantation, single-cell electroporation was performed under isoflurane anesthesia (4% for induction, then 1%). The body temperature was controlled and maintained at 37°C. At least 1 h before the surgery, the mice were injected with dexamethasone (5 mg/ml, 200 μl per mouse, intramuscular, Helvepharm, Zentiva) and just before the surgery with Carprofen (0.3 ml at 0.5 mg/ml, i.p., Rimadyl, Pfizer). The mice were head-fixed using the implanted metal head-post. The eyes were protected with a hydrating eye gel (VITA-POS, Pharma Medica AG). First, a circular craniotomy of around 3.5 mm was drilled around the center of the C2 barrel column using the blood vessel map. Depending on the mouse, a full durotomy was sometimes performed in order to facilitate access to the cortex. *In vivo* shadow single-cell electroporation was targeted to layer 2/3 neurons using a two-photon microscope (Judkewitz et al., 2009; Pala and Petersen, 2015). Glass capillary pipettes with resistances of 10–17 MΩ were filled with intracellular solution containing (in mM): 135 potassium gluconate, 4 KCl, 10 HEPES, 10 sodium phosphocreatine, 4 MgATP, 0.3 Na_3_GTP (adjusted to pH 7.3 with KOH) into which 100 μM Alexa (Thermofisher Scientific, A10436) and pCAG-EGFP (Addgene, 11150) plasmids were added (final plasmid concentration at 200 ng/μl). During the electroporation, the pipette was inserted into the cortex while continuously reading the 3D position of the pipette tip using a micromanipulator (SM7-Luigs and Neumann). Layer 2/3 was identified by vertical subpial depth (at least 150 μm from pia surface) and by a sudden increase in cell density. After close contact to a randomly chosen layer 2/3 neuron (increase of the pipette resistance by 20%), 50 pulses of negative voltage steps (0.5 ms, −12 V) were delivered at 50 Hz using a pulse generator (Axoporator 800A, Molecular Devices). After the electroporation, the pipette was slowly retracted and the immediate visual appearance of the neuron was used to judge the success of the procedure (i.e., if the cell remained intact and filled with the intracellular dye). Typically, two-three cells were electroporated per animal. At the end of the procedure a triple glass window assembly, consisting of a 5 mm diameter and two 3 mm diameter coverslips of #1 thickness (CS-3R, Warner Instruments), was placed over the craniotomy and fixed on the skull using UV-curing adhesive (Thorlabs, NOA68). The mouse was then returned to its home-cage and was allowed to recover for at least 3 days. The expression of the GFP in the electroporated neurons was evaluated 3–5 days after the procedure, through the cranial window, using an epi-fluorescent microscope and/or the two-photon microscope. Only animals with a single expressing cell were selected for the following steps while animals with multiple cells were excluded. On average approximately one-quarter of the electroporated mice contained a single brightly labeled neuron.

### Sample Preparation and Two-Photon Tomography

After single-cell expression of the fluorescent protein, the mice were transcardially perfused under deep anesthesia (pentobarbital 150 mg/kg, i.p.) using 4% paraformaldehyde (PFA) diluted in PBS (Electron Microscopy Science, USA), the brains were extracted and post-fixed in PFA overnight. After post-fixation, the brain tissue underwent a passive clearing procedure using a modified-CUBIC (mCUBIC) solution (Susaki et al., 2015) consisting of 25% w/w N,N,N’,N’-tetrakis(2-hydroxypropyl)ethylenediamine, 15% w/w of Triton-X and 60% w/w of dH_2_O. Whole brains were firstly incubated in mCUBIC solution at 37°C in a shaker (at 90 RPM) for 4 days. On day 4, the solution was replaced with a fresh one and the incubation continued for another 4 days. After the clearing process, the tissue was washed three times for 1 h using 50 mM phosphate-buffered (PB) solution. For 3 out of the 10 animals the cleared brain tissue was then incubated in 5% gelatin (Sigma G1890) for 2–4 h at 37°C (for the rest of the brains this step was omitted as it did not seem to improve imaging quality). Finally, the brains were placed in 4% PFA for 24–36 h at 4°C to cross-link and then washed with 50 mM PB. In order to increase the stability of the tissue during serial section two-photon tomography, the tissue was embedded in 5% agarose (Type-I agarose, Merck KGaA, Germany, A6013).

Whole brain 3D imaging was performed using a custom-made two-photon serial sectioning microscope which was controlled by the MATLAB-based software ScanImage 2017b (Vidrio Technologies, USA, for the 2P imaging) and BakingTray^1^ (for the serial sectioning). In summary, the imaging setup consists of a 2P microscope coupled with a vibratome head (VT1000S, Leica, Germany) and an X/Y/Z high precision stage (X/Y: V-580; Z: L-310, Physik Instrumente, Germany), similar to previously described (Han et al., 2018). The vibratome was set to slice the brain at 50 μm physical slice thickness, and 10 optical sections per physical section were acquired using a high-precision piezo objective scanner (PIFOC P-725, Physik Instrumente, Germany). A 16× water immersion objective was used with a resolution of 0.8 μm in X and Y and measured axial point spread function (PSF) at ~5 μm full width at half maximum. We collected fluorescence in the green channel (500–550 nm, ET525/50) and each section consisted of 1,025 × 1,025 μm tiles overlapping at 7%. The final voxel size was 0.8 × 0.8 × 5 μm (X, Y, Z).

After acquisition, the raw tiles were stitched using the MATLAB-based package StichIt^2^. This software applies illumination correction based on the average tile in each optical plane and stitches the tiles based on the actual position in 3D, as registered by the high precision motors.

### Tracing of Axons and Dendrites

After stitching, the data were tera-converted using the Vaa3D-Terafly software suite and the whole brain was visualized in 3D at different scales^3^. Subsequently, we used the Vaa3D module for software-assisted neuron tracing in order to place nodes in 3D. The placement of the nodes was based on the fluorescent signal in the image. The final output of Vaa3D was a .eswc file containing thousands of rows, each one consisting of node ID, x, y, z coordinate, radius value, neurite type, and parent node ID.

### Extracting Barrel Column Masks From the Digital Atlas

We used ilastik v1.3.3 (Berg et al., 2019) to segment the barrel columns from the grayscale anatomical image of the Allen Common Coordinate Framework version 3 (CCF, Wang et al., 2020). In ilastik, a human annotator labeled a few example pixels as the barrel column which was used to train a classifier to segment the entire image stack. Then the resultant mask image stack was used as a brain atlas parcellation file.

### Registration to a Digital Atlas

The stitched brain slices and the annotated neurons were registered to the Allen CCF version 3 (Wang et al., 2020) using a Python custom-written script, inspired by the MATLAB-based ARA tools^4^. At the first step, the data were down-sampled in X, Y, Z in order to match the 25 × 25 × 25 μm voxel size of the CCF. Next, the open source medical image registration suite Elastix^5^ was utilized in order to register the grayscale CCF anatomical image to the acquired brain slices in 3D, using rigid, affine, and nonrigid transformations. Once this transformation was computed, it was also applied to the parcellation file of the CCF and thus every voxel of the imaged brain data was assigned with a brain area ID matching to a unique brain area. Finally, we apply the same transformation to the barrel column mask file for the location of the neuron and visualizations.

### Quantification of Neurite Length

For the quantification of the neurite length, the x, y, z node coordinates of the Vaa3D .eswc files were transformed to physical distances in μm using the imaging resolution values (0.8 μm in X and Y and 5 μm in Z). Afterward the Vaa3D resampling plugin was used^6^ to resample the annotation points and space them equally every 1 μm. Finally, the number of points in every brain area was counted and transformed in neurite length in μm. If the annotation node of a neurite coincided with a given voxel, that node was assigned to the brain area corresponding to this voxel according to the CCF parcellation file.

### Immunohistochemistry

In order to test for the presence of additional axonal arborizations that might not have been resolved in the two-photon tomography, we collected the 50 μm-thick slices immediately after two-photon tomographical imaging. We then amplified the GFP signal with immunostaining. During this process, the slices were firstly incubated in blocking buffer 0.3% Triton (Applichem, Germany) and 2% normal goat serum (NGS, Vector, S-1000-L020) in PBS (0.9% NaCl, 0.01 M phosphate buffer, pH 7.4) for an hour. Then, we incubated the sample for 48 h shaking at 4°C in the primary anti-GFP antibody (rabbit polyclonal 1:5,000, Abcam 290, UK) together with 0.3% Triton X-100 in PBS, followed by two washes with PBS for 10 min. Subsequently, the slices were placed in the secondary antibody (goat anti-rabbit conjugated to Alexa 488 1:200, Life Technologies A-11012) together with 0.3% Triton X-100 in PBS for 2–2.5 h at room temperature. At the final step, the slices were washed in PBS three times for 10 min and mounted on Superfrost slides using, 4-Diazabicyclo[2.2.2]octane (DABCO, Sigma-Aldrich D27802, USA) as mounting medium. Images of the stained sections were obtained using the two-photon tomography microscope at the same laser power levels as the original imaging, enabling direct comparison.

### Data Availability

The data and code are freely available in the Open Access CERN database Zenodo: https://doi.org/10.5281/zenodo.5813321.

## Results

### Reconstruction of Dendrites and Axons of Single Neurons in Layer 2/3 Barrel Cortex

**Figure 1:**
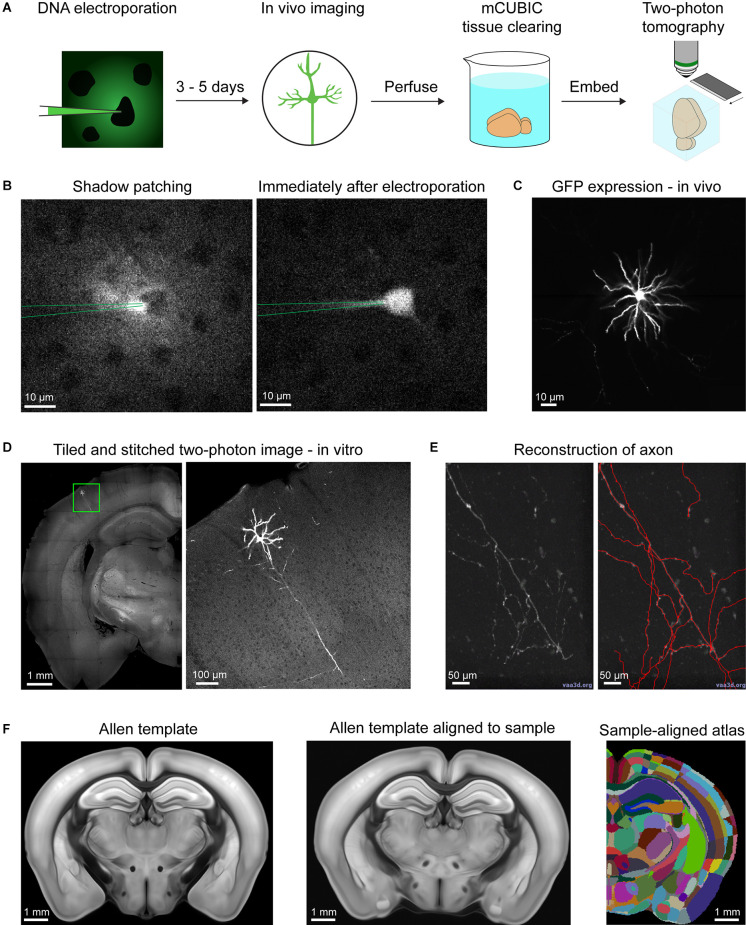
Acquisition and analysis pipeline for single-cell reconstruction. **(A)** Shadow electroporation labels a single neuron by introducing GFP plasmids. After 3–5 days of expression time, the cell was viewed through a cranial window under a two-photon microscope to control for GFP expression and the absence of any sign of apoptosis. Animals were then perfused and the brain was cleared in a modified CUBIC solution. Finally, the sample was embedded in agarose and imaged under a two-photon tomographic microscope. **(B)** Example snapshots of an electroporation session. *Left*, fluorescent dye from the electroporation pipette fills the extracellular space revealing cell bodies as shadows in the image. The pipette approaches and contacts a randomly selected neuron. *Right*, following electroporation, the fluorescent dye rapidly fills the cell indicating successful pipette content delivery into the cell. **(C)** Quality check of the labeled neuron through the cranial window before perfusion. **(D)** Example image acquired from two-photon tomography. *Left*, a coronal section with a region of interest near the cell body. *Right*, the region of interest at higher resolution, showing the cell body, its dendrites, and its main descending axon. **(E)** Semi-automatic neuron reconstruction using Vaa3d. *Left*, an example region with axon in Vaa3d. *Right*, the same region with annotations (red) highlighting the axon. **(F)** Alignment with the Allen Mouse CCFv3. *Left*, a coronal section of the CCFv3 template. *Middle*, the template deformed to align with the sample space. *Right*, the CCFv3 atlas deformed in the same way as the template to match the sample space.

In order to reconstruct neuronal arborizations of single neurons in layer 2/3 of the primary somatosensory cortex barrel field (SSp-bfd), we performed “shadow” single-cell electroporation *in vivo* under the guidance of a two-photon microscope (Judkewitz et al., 2009; Figures 1A,B). A glass pipette filled with intracellular solution, fluorescent dye, and GFP DNA plasmids was inserted through a craniotomy into layer 2/3 of the SSp-bfd and positioned in close contact to the cell membrane of a randomly chosen cell in our field-of-view (Figure 1B). Afterward, a train of negative electrical pulses was delivered in order to transiently rupture the cell’s membrane, permitting the entrance of the pipette solution into the cell’s cytoplasm. If the electroporation procedure was successful, the cell was immediately filled with the fluorescent dye and remained intact after pipette retraction. After 3–5 days, a quality check of the cell’s health and the expression levels of GFP was performed under the two-photon microscope through a cranial window (Figure 1C). If only a single neuron per mouse expressed GFP and it did not show any signs of dendritic “blebbing” or cell death (Batista Napotnik et al., 2021), the animal was transcardially perfused with PFA in order to fix the brain. Subsequently, the extracted brain was partially cleared using mCUBIC (Susaki et al., 2015) and prepared for two-photon serial section tomographic 3D imaging (Han et al., 2018; Figure 1D). After imaging the whole brain, individual tiles were computationally stitched to reassemble full brain slices which were then imported into Vaa3D software for semi-automatic annotation of the neuronal structures, based on the GFP fluorescence signal (Figures 1D,E). Following annotation, the tomographic structural images were used for registration to the Allen Mouse Brain Common Coordinare Framework (CCF). Once this step was completed, the location of the different neuronal arborizations in various brain areas was assessed for each of the 10 reconstructed neurons in this study, using the Allen Mouse Brain standardized parcellations (Figure 1F, Supplementary Figure 1, and Supplementary Table 1).

Importantly, we do not think that our anatomical reconstructions are complete because anti-GFP immunolabeling to enhance the signal-to-noise ratio of axonal fluorescence revealed additional axon not found through two-photon tomography (Supplementary Figure 2). The anatomical reconstructions presented in this study therefore only reveal a portion of the full extent of the axons, but nonetheless provide important information characterizing their apparent diversity.

### Diverse Axonal Projections of Single Neurons in Layer 2/3 of wS1

**Figure 2:**
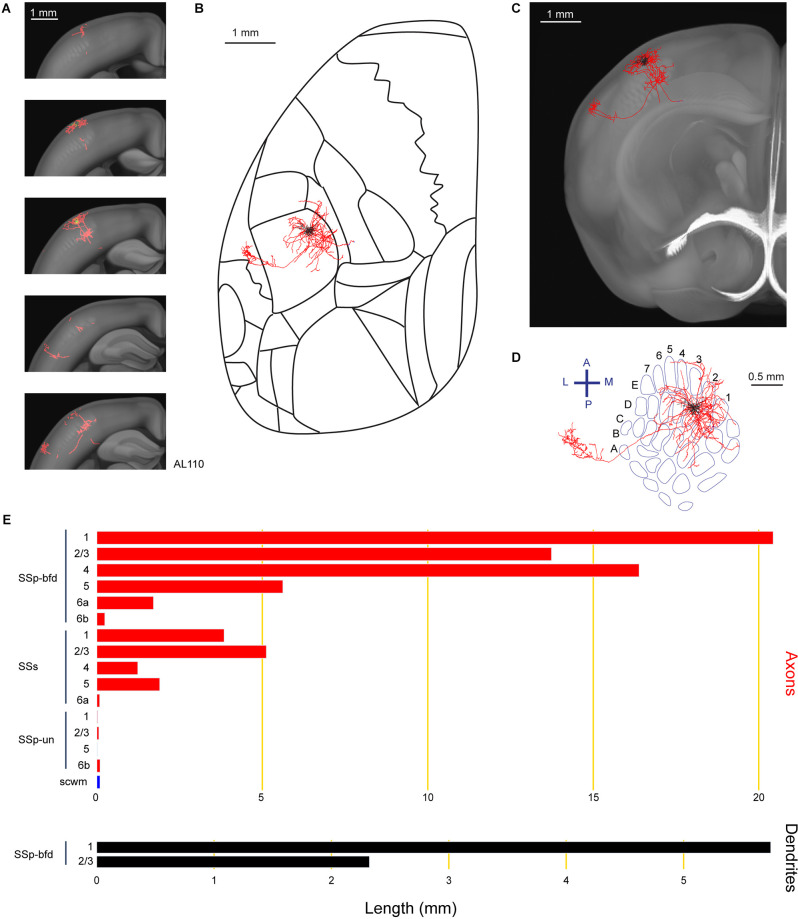
Reconstruction and quantification of example neuron AL110 with projections to the supplementary somatosensory cortex. **(A)** Serial overlays of GFP-labeled axon (red) and dendrites (green) in coronal views encompassing the anterior-posterior span of the axons of 1.5 mm. Each section represents a maximum projection of 300 μm. **(B)** Maximum projection of the reconstructed axon and dendrites in horizontal view, aligned to the Allen Mouse CCFv3 to indicate the boundaries between cortical regions. **(C)** Maximum projection of reconstructed axon and dendrites in coronal view overlaid with an anatomical section from the Allen Mouse CCFv3. **(D)** Maximum projection of reconstructed axon and dendrites in a tangential view (rotated 30 degrees) over the barrel field (blue). The cell is located in the D3 barrel column. **(E)** Quantification of axonal (*top*) and dendritic (*bottom*) length in respective brain regions identified by the Allen Mouse CCFv3. For **(B)** to **(E)**: dendrites are shown in black, axon in gray matter is shown in red, and axon in white matter is shown in blue.

Previous bulk anterograde labeling of long-range axons of neurons with somata in SSp-bfd showed a prominent projection target in the secondary somatosensory cortex (SSs; White and DeAmicis, 1977; Welker et al., 1988; Aronoff et al., 2010; Yamashita et al., 2018). In this study, we also found a layer 2/3 neuron (AL110) with a prominent axonal arborization in SSs (Figure 2). The soma of this neuron was located in the D3 barrel column. The neuron had a total length of 70.7 mm of axon and 8.1 mm of dendrites. The majority of the axon extended within the granular and supragranular layers of SSp-bfd, although the layer assignments should be interpreted with caution due to potential registration errors and limitations in the digital atlas. A part of the axon extended to SSs, in particular in layer 1 and layer 2/3. In agreement with previous studies (Frostig et al., 2008; Stehberg et al., 2014; Yamashita et al., 2018), the long-range axonal branch connecting SSp-bfd and SSs traveled within the cortical gray matter without entering white matter fiber tracts. In addition to innervating SSs, a small portion of its axon also projected to an unassigned region in the primary somatosensory cortex (SSp-un) and the supra-callosal white matter (scwm).

**Figure 3:**
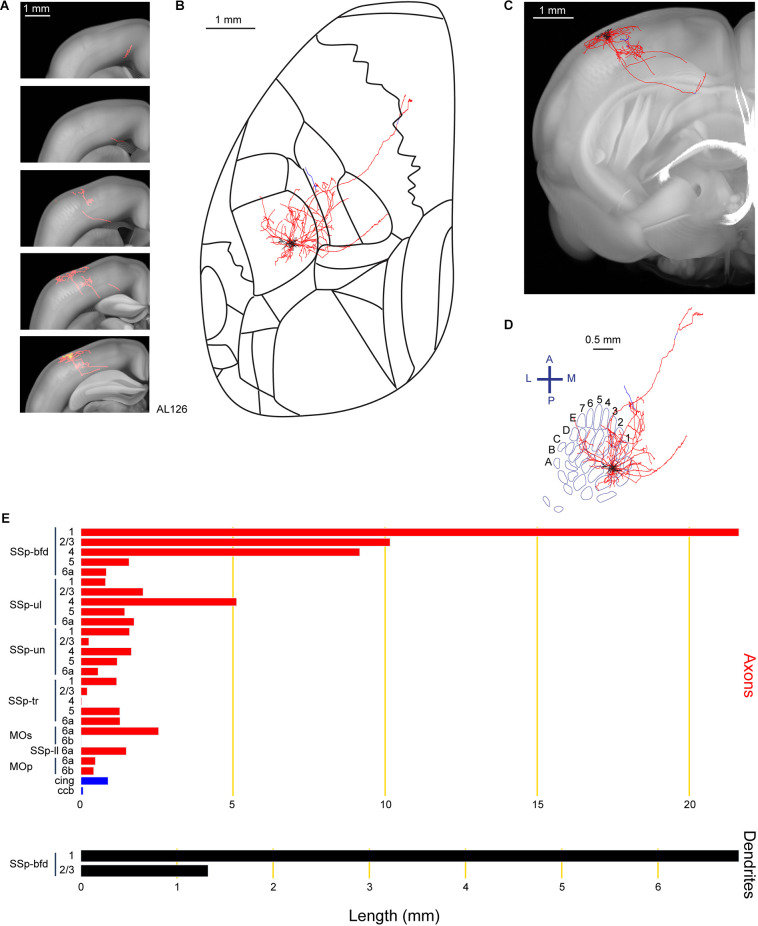
Reconstruction and quantification of example neuron AL126 with projections to the primary and secondary motor cortex. **(A)** Serial overlays of GFP-labeled axon (red) and dendrites (green) in coronal views encompassing the anterior-posterior span of the axons of 3.5 mm. Each section represents a maximum projection of 700 μm. **(B)** Maximum projection of the reconstructed axon and dendrites in horizontal view. **(C)** Maximum projection of the axon and dendrites in coronal view. **(D)** Maximum projection of the axon and dendrites in tangential view (rotated 30 degrees) over the barrel field. The neuron is located in the D1 barrel column. **(E)** Quantification of axonal (top) and dendritic (bottom) length in respective brain regions identified by the Allen Mouse CCFv3. For **(B)** to **(E)**: dendrites are shown in black; axon in neocortical gray matter is shown in red; axon in white matter is shown in blue.

Another important projection target of layer 2/3 neurons is the primary motor cortex (MOp; Aronoff et al., 2010; Yamashita et al., 2013, 2018; Chen et al., 2015; Yamashita and Petersen, 2016; Vavladeli et al., 2020). In this study, we also found a neuron (AL126) with axon in MOp and secondary motor cortex (MOs; Figure 3). Similar to neuron AL110, and in agreement with previous studies (Frostig et al., 2008; Stehberg et al., 2014; Yamashita et al., 2018), the long-range axonal branch connecting SSp-bfd and MOp/MOs traveled within the cortical gray matter without entering white matter fiber tracts. This neuron, with 8.2 mm of dendrites was situated in the D1 barrel column. With a total axonal length of 69.8 mm, this neuron extended its axon primarily within SSp-bfd, but also to other primary sensory areas such as the upper limb (SSp-ul), trunk (SSp-tr), lower limb (SSp-ll) and SSp-un. One axonal branch of this neuron traveled within the white matter fiber bundle system (cingulum bundle, cing, and the corpus callosum body, ccb).

**Figure 4:**
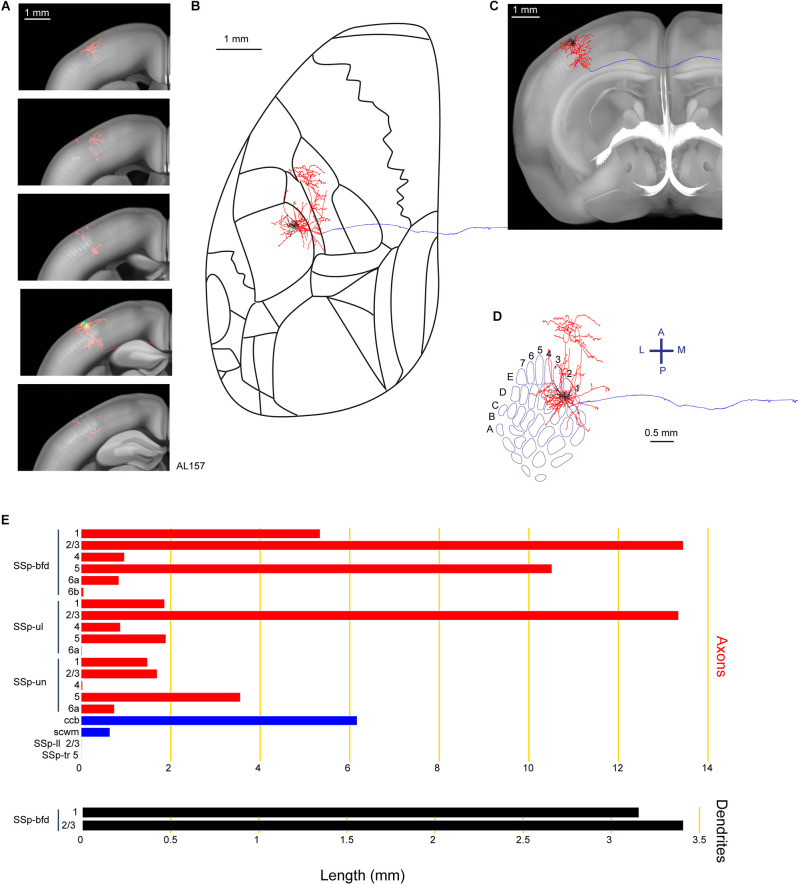
Reconstruction and quantification of example neuron AL157 with projections to the primary somatosensory cortex upper limb area. **(A)** Serial overlays of axon (red) and dendrites (green) in coronal views encompassing the anterior-posterior span of the axons of 2.125 mm. Each section represents a maximum projection of 425 μm. **(B)** Maximum projection of the reconstructed axon and dendrites in horizontal view. **(C)** Maximum projection of the axon and dendrites in coronal view. **(D)** Maximum projection of the axon and dendrites in tangential view (rotated 30 degrees) over the barrel field. The cell is located in the E2 barrel column. **(E)** Quantification of axonal (top) and dendritic (bottom) length in respective brain regions identified by the Allen Mouse CCFv3. For **(B)** to **(E)**: dendrites are shown in black; axon in neocortical gray matter is shown in red; axon in white matter is shown in blue.

Among our reconstructed neurons, we found neuron AL157 with a long-range axonal projection largely targeting the SSp-ul, consistent with previously-reported innervation patterns from bulk labeling of rat and mouse barrel cortex showing innervation of forelimb cortex (Zakiewicz et al., 2014; Zingg et al., 2014; Figure 4). The axons of this neuron also extend to the SSp-un in the anterior-medial proximity of the barrel field. Another major branch of the axon travels in the callosal fiber bundle system (ccb and scwm), entering the contralateral hemisphere. However, we were unable to identify any further extensions of this callosal axonal branch with our current protocol. In total, we found 6.6 mm of dendrite and 63.5 mm of axon for this neuron located in the E2 barrel column.

**Figure 5:**
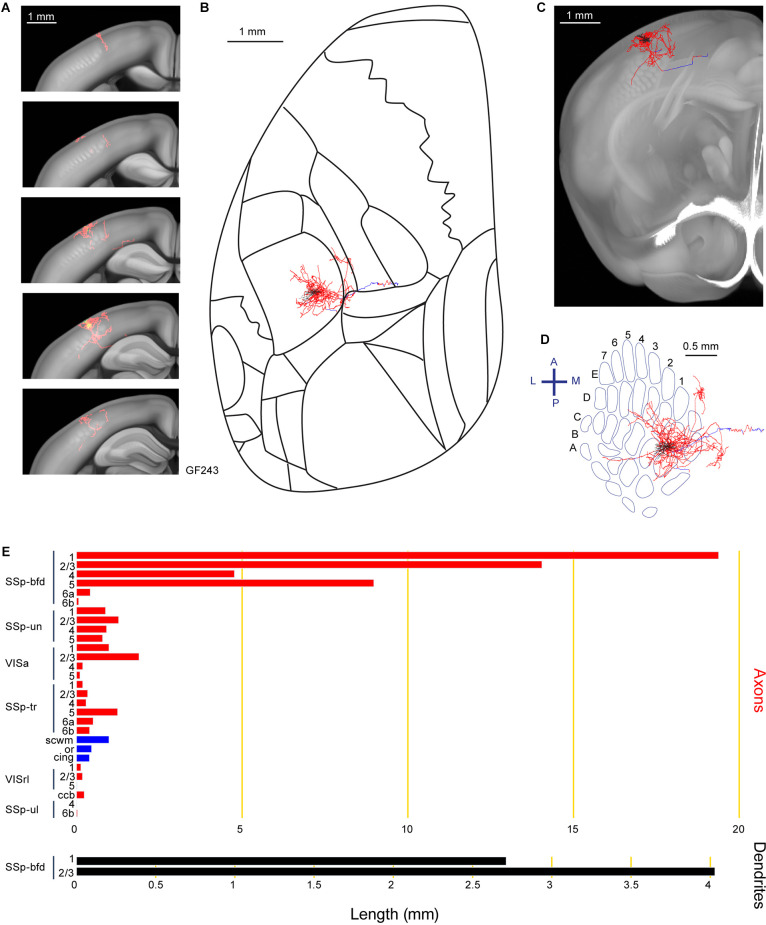
Reconstruction and quantification of example neuron GF243 with projections to an unassigned region of the primary somatosensory cortex and an anterior visual area. **(A)** Serial overlays of axon (red) and dendrites (green) in coronal views encompassing the anterior-posterior span of the axons of 1.25 mm. Each section represents a maximum projection of 250 μm. **(B)** Maximum projection of the reconstructed axon and dendrites in horizontal view. **(C)** Maximum projection of the axon and dendrites in coronal view. **(D)** Maximum projection of the axon and dendrites in tangential view (rotated 30 degrees) over the barrel field. The cell body is in the septa between the C1 and D1 barrel columns. **(E)** Quantification of axonal (top) and dendritic (bottom) length in respective brain regions identified by the Allen Mouse CCFv3. For **(B)** to **(E)**: dendrites are shown in black; axon in neocortical gray matter is shown in red; axon in white matter is shown in blue.

Axonal arborizations located in SSp-un were common across several neurons in our current study, including neuron GF243 with a highly localized columnar innervation pattern in SSp-un, as well as innervating VISa (Figure 5). We identified 60.1 mm of axons and 6.7 mm of dendrite for this neuron situated in the septa between the C1 and D1 barrel columns. Other target areas for this neuron includes SSp-tr, VISrl, fiber bundle systems (cingulum bundle, cing, scwm, and ccb) and SSp-ul.

**Figure 6:**
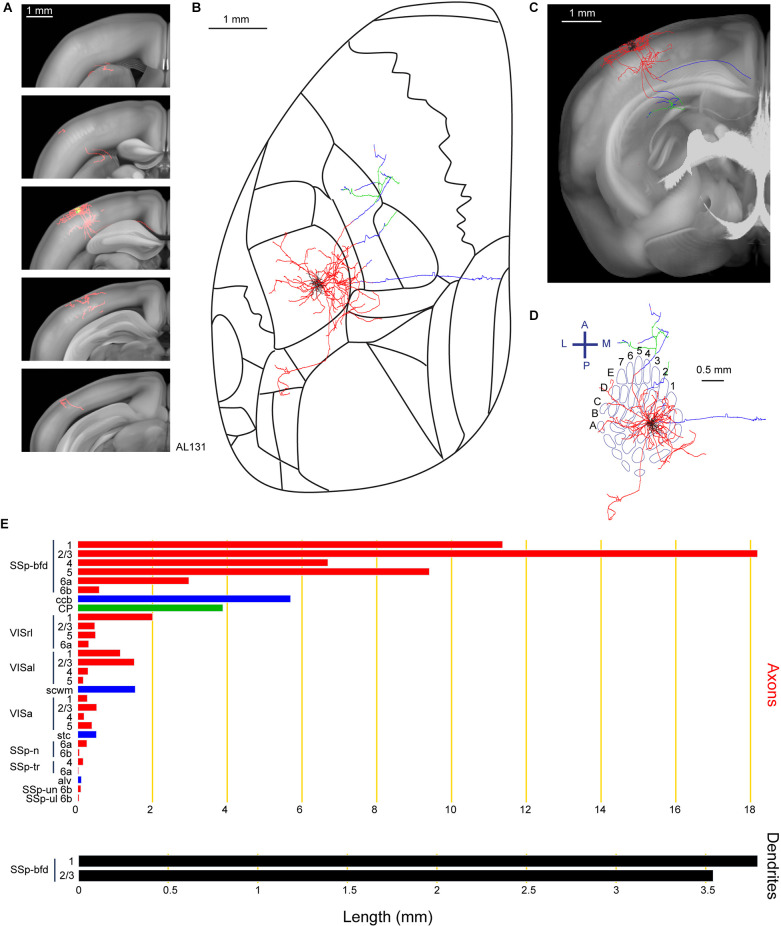
Reconstruction and quantification of example neuron AL131 with projections to the caudoputamen and multiple visual areas. **(A)** Serial overlays of axon (red) and dendrites (green) in coronal views encompassing the anterior-posterior span of the axons of 4.125 mm. Each section represents a maximum projection of 825 μm. **(B)** Maximum projection of the reconstructed axon and dendrites in horizontal view. **(C)** Maximum projection of the axon and dendrites in coronal view. **(D)** Maximum projection of the axon and dendrites in tangential view (rotated 30 degrees) over the barrel field. The neuron is in the C2 barrel column. **(E)** Quantification of axonal (top) and dendritic (bottom) length in respective brain regions identified by the Allen Mouse CCFv3. For **(B)** to **(E)**: dendrites are shown in black; axon in neocortical gray matter is shown in red; axon in the striatum is shown in green; axon in white matter is shown in blue.

Among the subcortical projections of wS1 layer 2/3 excitatory neurons, is the dorsolateral striatum (Sippy et al., 2015; Yamashita et al., 2018). Consistent with those findings, neuron AL131 had prominent axonal targets in the dorsolateral part of the caudoputamen (CP; Figure 6). A total length of 69.2 mm of the axon was annotated for this neuron as well as 7.3 mm of dendrites located in the C2 barrel column. In addition to the CP, axons were identified in multiple visual areas such as the rostrolateral areas (VISrl), anterolateral areas (VISal) and anterior areas (VISa), as well as several regions in the primary somatosensory area (nose, SSp-n, SSp-tr, SSp-un, and SSp-ul). This neuron also has a prominent branch traveling in the callosal fiber bundle system (scwm for this specific case).

**Figure 7:**
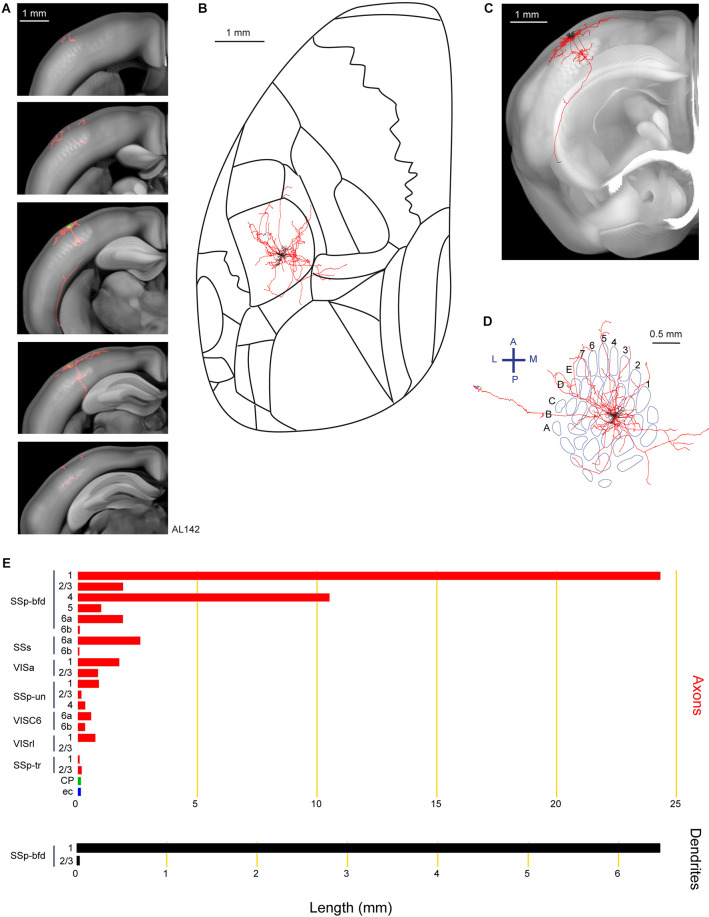
Reconstruction and quantification of example neuron AL142 with projections to the lateral caudoputamen and towards the amygdala. **(A)** Serial overlays of axon (red) and dendrites (green) in coronal views encompassing the anterior-posterior span of the axons of 2.375 mm. Each section represents a maximum projection of 475 μm. **(B)** Maximum projection of the reconstructed axon and dendrites in horizontal view. **(C)** Maximum projection of the axon and dendrites in coronal view. **(D)** Maximum projection of the axon and dendrites in tangential view (rotated 30 degrees) over the barrel field. The cell is in the septa between the D2 and D3 barrel columns. **(E)** Quantification of axonal (top) and dendritic (bottom) length in respective brain regions identified by the Allen Mouse CCFv3. For **(B)** to **(E)**: dendrites are shown in black; axon in neocortical gray matter is shown in red; axon in the striatum is shown in green; axon in white matter is shown in blue.

Lastly, we show an example neuron (AL142) extending a long axonal branch laterally to the CP, apparently heading towards the most dorsal aspect of the amygdala (Figure 7). In comparison to neuron AL131 innervating the dorsolateral striatum (Figure 6), the axons of neuron AL142 traversed deeper layers of SSp-bfd, SSs, Visceral area (VISC), external capsule (ec), with our tracing ending in the lateral parts of the CP near the most dorsal aspect of the amygdala (Figure 7). Other target areas for this neuron include the VISa, SSp-un, VISrl and SSp-tr. For this neuron, we did not identify any signs of axon within the callosal fiber bundle system. This neuron had a total of 48.8 mm of axon and 6.5 mm of dendrites and its cell body was located in the septa between the D2 and D3 barrel columns.

### Summary of All SSp-bfd Layer 2/3 Neurons Reconstructed

**Figure 8:**
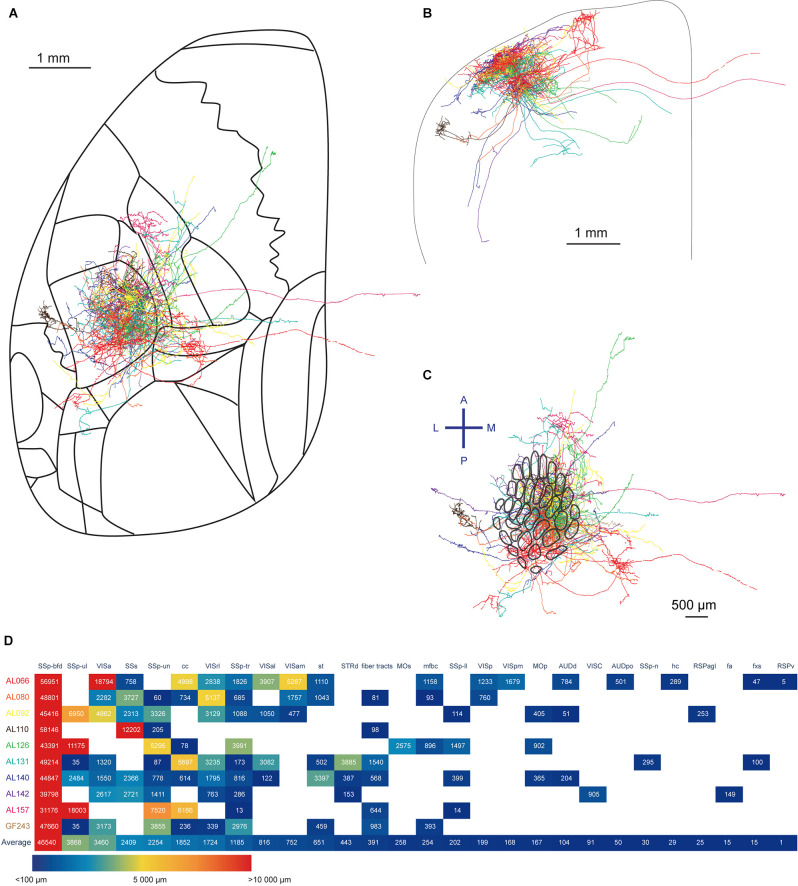
Summary of the ten reconstructed neurons. **(A)** Horizontal overlay of axons aligned to the Allen Mouse CCFv3. The axon of each neuron is shown in a different color. **(B)** Coronal overlay of axons. **(C)** Tangential view of axons aligned to the barrel map. **(D)** Quantification of axonal length in respective brain regions. The length of the axon in each layer of a specific region is summed up. The different brain regions receiving projections from the labeled neurons are sorted according to the mean length of the reconstructed axon across all neurons in each region of interest, from the longest axonal length on the left to the shortest axonal length on the right.

Finally, we summarize the 10 neurons reconstructed in this study (Figures 2–7 and Supplementary Figures 3–7) and overlay them in order to get an idea of the overall projection profile (Figure 8). We quantified axonal length in each brain region identified summing across layers and subregions (Figure 8D). Most of the axonal length resided within the SSp-bfd. It is interesting to note that SSp-ul and VISa ranked second and third, then followed by the SSs and SSp-un as fourth and fifth. Although ranked lower in the average amount of axons, nine out of the 10 neurons projected to SSp-tr. Other target regions include MOp and MOs, dorsal region of the striatum (STRd), several other SSp regions (SSp-tr, SSp-ll), various visual areas (for instance, VISam, VISp, and VISpm), multiple fiber bundles (stria terminalis, st, fiber tracts, cerebrum related, mfbc, hippocampal commissures, hc, corpus callosum anterior forceps, fa, and fornix system, fxs), retrosplenial areas (lateral agranular part, RSPagl and ventral part, RSPv), auditory areas (such as the dorsal auditory area AUDd and posterior auditory areas, AUDpo), and VISC. Seven of the 10 reconstructed neurons had axonal branches in the corpus callosum (cc) with none of these extending outside of the fiber tracts on the opposite hemisphere (Han et al., 2018; Yamashita et al., 2018).

## Discussion

We performed two-photon guided *in vivo* “shadow” electroporation to label single neurons in layer 2/3 of mouse barrel cortex by expression of GFP (Judkewitz et al., 2009). Through two-photon tomographic imaging and three-dimensional neuron reconstruction (Han et al., 2018) in relation to a digital mouse brain atlas, we quantified long-range projection regions among the 10 reconstructed cells finding a high degree of diversity.

### Diverse Projection Areas of Individual Neurons in Layer 2/3 Barrel Cortex

Among the literature, numerous reports have studied the SSp-bfd using broader approaches involving viral injections or other anterograde tracers that label thousands of neurons. Less is known on the finer scale investigation of individual neurons, but it seems likely that each neuron only projects to a subset of the regions that have been identified from bulk labeling approaches (Guo et al., 2017; Yamashita et al., 2018).

Projections between the SSp-bfd to the SSs have been shown to be heavily reciprocal, and similar observations between the SSp-bfd and the motor regions (MOp and MOs) have been reported (Aronoff et al., 2010; Mao et al., 2011; Zingg et al., 2014). Here, we further report SSp-bfd axons also project to other parts of the SSp such as the upper limb, lower limb, trunk, and unassigned regions. Projections to these somatosensory regions have been previously reported in the Mouse Connectome project where an anterograde and a retrograde tracer were co-injected into a single area (Zingg et al., 2014), showing reciprocal projections. Such organization likely aids the integration of sensory information across the somatotopic map. The extensive axonal arborization within SSp-bfd is likely important for integrating sensory information across the whisker pad, necessary for determining object shape (Brown et al., 2021; Rodgers et al., 2021). The important projection to SSp-ul might be important during running where the ventral whiskers touch the ground before the forepaw as if to ensure safe placement of the paw during locomotion (Grant et al., 2018). Similar to previous studies (Frostig et al., 2008; Stehberg et al., 2014; Yamashita et al., 2018), we found that the long-range axonal projections typically traversed cortical boundaries traveling in the neocortical gray matter rather than entering the white matter fiber tracts. The horizontally extending axons are likely to contribute to the large functional spread of signals evoked by the deflection of even just a single whisker (Ferezou et al., 2007; Frostig et al., 2008; Johnson and Frostig, 2016, 2018).

It has also been demonstrated that there is heterogeneity in the projection pattern within the SSp-bfd (Zingg et al., 2014). The caudal-medial barrel field (cm-bfd) projects to SSp-tr and SSp-ll while the antero-lateral barrel field (al-bfd) showed a preference for SSp nose and mouth regions. With our current study, all labeled neurons are located near the B, C, D, or E rows with arc position 1, 2, or 3, which are relatively caudal and medial in the barrel field (Figure 8C). In agreement with previous suggestions, our results show all neurons except for AL110 send their axons to the SSp-tr. Furthermore, anterograde tracers injected in the cm-bfd, but not the al-bfd, have also been found to label axonal projections in the AUDd, AUDp and AUDv (Zingg et al., 2014). Projections to the AUDd and AUDp were also observed in the current study (Figures 8A,D). In addition, anterograde tracers injected in the cm-bfd, revealed axons in the VISC, but this is not observed in those with al-bfd injections (Zingg et al., 2014). Being the only neuron with axons identified in VISC in this study, AL142 is located near the D2 barrel which takes a relatively central-medial position in the posterior barrel field (Figure 6). Additional reconstructions of single neurons are necessary to make more in-depth comparisons to quantifications obtained from viral labeling.

One of the common projection targets of our reconstructed neurons is the unassigned SSp-un region. In the present study, nine out of 10 neurons showed axons in SSp-un and it is ranked having the fourth greatest amount of axon among all regions identified (Figure 8D). Projections in the same region have also been reported in the Allen Mouse Brain Connectivity Atlas (Oh et al., 2014) in a particular experiment (Experiment 298718778) where a Cre-dependent anterograde tracer was injected to SSp-bfd of a Rasgrf2-dCre mouse that labels mostly layer 2/3 neurons. This region, immediately medial to the SSp-bfd, corresponds to the dysgranular zone that has been reported in both rats and mice (Koralek et al., 1990; Lee and Kim, 2012; Yamashita et al., 2018). Neurons within this region send their axons to the striatum, thalamus, and midbrain (Lee and Kim, 2012). In line with previous studies which indicated that there are “hot spots” for axons extending to the dysgranular zone (Yamashita et al., 2018), we also identify a large number of axons in this region. Given the frequency of occurrence in single cell projection profiles and the number of axons it receives, the SSp-un may be an important region to investigate in future experiments.

Additionally, several neurons had axons projecting to higher order visual related areas. These types of projections have been previously reported for both the rat and the mouse barrel cortex and are likely to be reciprocal (Zakiewicz et al., 2014; Zingg et al., 2014; Yamashita et al., 2018). One possible hypothesis for the role of this connectivity between SSp and visual areas is multisensory integration (Zakiewicz et al., 2014).

Neurons projecting to MOp and to SSs might form two distinct populations both anatomically, functionally, and genetically (Chen et al., 2013, 2015; Yamashita et al., 2013, 2018; Sorensen et al., 2015; Yamashita and Petersen, 2016). In previous work (Yamashita et al., 2018), retrograde tracers were injected to SSs or MOp, and neurons in the SSp-bfd labeled with these tracers were selectively targeted for electroporation. Axonal reconstructions suggested that neurons projecting to SSs (S2p) do not project to MOp, while the MOp projecting neurons (M1p) projected only weakly to SSs. Consistent with this, in the small sample of neurons in the current study, the strong projectors to MOp and MOs (AL126) and to SSs (AL110) indeed seem to be non-overlapping in their axonal arborizations (Figure 8D). However, there are also neurons that project some axons to both regions (Supplementary Figures 5, 6). Further studies with greater numbers of single-neuron reconstructions may provide more accurate depictions of neuron categories based on anatomical information. Given that the M1p and S2p neurons were distinct both functionally and genetically (Yamashita et al., 2013; Sorensen et al., 2015), future studies might reveal more categories of layer 2/3 neurons such as VISam-projecting or STRd-projecting neurons.

### Axons in the Fiber Bundles

Axons in fiber bundles heading toward the contralateral hemisphere were frequently found among our reconstructed neurons. While 7 out of 10 neurons showed axons within the corpus callosum, none continued to exit. Several other studies that aimed to reconstruct single neurons have also reported a paucity of axons in the contralateral hemisphere (Han et al., 2018; Yamashita et al., 2018). Viral-based anatomical studies show some axons extending within the corpus callosum, winding past the midline and exiting to regions such as the contralateral SSp-bfd (Zingg et al., 2014; Yamashita et al., 2018). A study on developmental refinements of callosal projections in the SSp-bfd showed that although layer 2/3 neurons do show eliminations of contralateral projecting axons, this process stabilizes around postnatal day 15 (De León Reyes et al., 2019). It might be that some of these axons did not fully retract and leaving segments still within the corpus callosum. Our current approach does not detect all axons (Supplementary Figure 2), and it is possible that future studies will reveal more innervation of the contralateral hemisphere by layer 2/3 neurons. As opposed to a detection-related issue, this may also arise from incomplete GFP diffusion. The dense fiber bundles may limit the diffusion of fluorescent proteins, resulting in incomplete filling of the axon (Yamashita et al., 2018). To resolve these questions, future viral-based anatomical studies with higher imaging resolution are required to compare the number of axons at different points of the trajectory (such as at the entrance of the corpus callosum vs. at the points of exit on the contralateral side). From a single cell labeling approach, further studies could also inject retrograde tracers to the SSp-bfd followed by targeted electroporation in the hemisphere contralateral to the injection and subsequent neuronal reconstruction.

### Limitations and Future Perspectives

A major limitation of the present work is the incompleteness of the reconstructed axonal arborizations. Further signal enhancement appears to be essential (Supplementary Figure 2) and follow-up studies will need to incorporate these considerations. Assuming that the labeling method (be it electroporation or viral injections) sufficiently fills up the neuron and its entire extensions, sample pre-processing involving signal amplifications would provide an important step toward the true anatomical representation. Several whole-brain volumetric imaging techniques in combination with signal amplification processes have been developed which could be helpful for future experiments (Renier et al., 2014; Gong et al., 2016; Wang et al., 2019; Winnubst et al., 2019). Nonetheless, the neurons reconstructed in this study have a total axonal length per neuron of 67.9 ± 13.0 mm (mean ± SD, *n* = 10), which is comparable to that of the only other study that we know of including layer 2/3 mouse barrel cortex neurons registered to the Allen atlas, which found a total axonal length per neuron of 49.9 ± 13.9 mm (mean ± SD, *n* = 9; Peng et al., 2021).

Anatomical investigations at single-neuron resolution provide valuable insights on where each neuron might send information. In addition to quantifying axonal length through GFP, in the future, it would be useful to also use a red fluorescent protein attached to presynaptic proteins in order to identify neurotransmitter release sites. To reveal network-level information while maintaining cellular resolution requires large amounts of single-neuron data. It is then possible to unravel patterns through clustering-based analysis and categorize projection types (Wang et al., 2019; Winnubst et al., 2019). The necessity for large data sets suggests the need to design high throughput methods for sample preparation, image acquisition, and axonal annotation. Currently, large parts of the published neuronal reconstructions are being done manually by human annotators, which not only require hours of labor work (Magliaro et al., 2019) but also are susceptible to human errors and/or biases. However, recent advances in machine learning and computer vision may accelerate the later part of the pipeline with minimal human supervision (Zhou et al., 2018; Huang et al., 2020; Li and Shen, 2020).

From a scientific perspective, the opportunity of labeling single neurons and recovering their morphology might help, in the future, to better determine the role of projection neurons in complex neural computations, such as reward-based learning. As previously mentioned, studies, where projection neurons were retrogradely labeled, have shown projection target-dependent neuronal activity, for example during goal-directed sensorimotor transformations (Chen et al., 2013, 2015; Yamashita and Petersen, 2016; Vavladeli et al., 2020). In those cases, an assumption of the projection area(s) to focus on was made beforehand in order to bulk inject the retrograde tracer. One can imagine that an unbiased and more refined experimental procedure could be followed during which the activity of different neurons can firstly be measured (for instance, using two-photon calcium or voltage imaging), followed by “activity-targeted” selection of neurons to be electroporated and reconstructed. This way, cells which show interesting activity patterns during learning or execution of different behavioral tasks could be labeled and their morphology and/or projection targets could be revealed. Although technically demanding, this type of experiments could provide unique datasets where morphology and function can be directly linked, shedding light on brain mechanisms that still remain unexplored.

## Data Availability Statement

The data and code are freely available in the Open Access CERN database Zenodo: https://doi.org/10.5281/zenodo.5813321.

## Ethics Statement

The animal study was reviewed and approved by Swiss Federal Veterinary Office (Canton of Vaud).

## Author Contributions

GF, YL, SC, and CP conceptualized the study. GF and YL obtained and analyzed data. GF, YL, and CP wrote the manuscript. CP provided overall supervision. All authors discussed and edited the manuscript. All authors contributed to the article and approved the submitted version.

## Conflict of Interest

The authors declare that the research was conducted in the absence of any commercial or financial relationships that could be construed as a potential conflict of interest.

## Publisher’s Note

All claims expressed in this article are solely those of the authors and do not necessarily represent those of their affiliated organizations, or those of the publisher, the editors and the reviewers. Any product that may be evaluated in this article, or claim that may be made by its manufacturer, is not guaranteed or endorsed by the publisher.

## Footnotes

^1^ https://github.com/SainsburyWellcomeCentre/BakingTray

^2^ https://github.com/SainsburyWellcomeCentre/StitchIt

^3^ http://home.penglab.com/proj/vaa3d/home/index.html

^4^ https://github.com/SainsburyWellcomeCentre/ara_tools

^5^ https://github.com/SuperElastix/elastix

^6^ https://github.com/Vaa3D/vaa3d_tools/tree/master/releasedplugins/v3dplugins/resampleswc

## References

[B1] Aronoff R. Matyas F. Mateo C. Ciron C. Schneider B. Petersen C. C. H. (2010). Long-range connectivity of mouse primary somatosensory barrel cortex. Eur. J. Neurosci. 31, 2221–2233. 10.1111/j.1460-9568.2010.07264.x 20550566

[B2] Batista Napotnik T. Polajžer T. Miklavčič D. (2021). Cell death due to electroporation—A review. Bioelectrochemistry 141:107871. 10.1016/j.bioelechem.2021.107871 34147013

[B3] Berg S. Kutra D. Kroeger T. Straehle C. N. Kausler B. X. Haubold C.. (2019). ilastik: interactive machine learning for (bio)image analysis. Nat. Methods 16, 1226–1232. 10.1038/s41592-019-0582-9 31570887

[B4] Brecht M. (2007). Barrel cortex and whisker-mediated behaviors. Curr. Opin. Neurobiol. 17, 408–416. 10.1016/j.conb.2007.07.008 17702566

[B5] Brown J. Oldenburg I. A. Telian G. I. Griffin S. Voges M. Jain V.. (2021). Spatial integration during active tactile sensation drives orientation perception. Neuron 109, 1707–1720.e7. 10.1016/j.neuron.2021.03.020 33826906 PMC8944414

[B6] Cajal S. R. Y. (1995). “Histology of the nervous system of man and vertebrates,” in History of Neuroscience, (New York: Oxford University Press)

[B7] Chen J. L. Carta S. Soldado-Magraner J. Schneider B. L. Helmchen F. (2013). Behaviour-dependent recruitment of long-range projection neurons in somatosensory cortex. Nature 499, 336–340. 10.1038/nature12236 23792559

[B8] Chen J. L. Margolis D. J. Stankov A. Sumanovski L. T. Schneider B. L. Helmchen F. (2015). Pathway-specific reorganization of projection neurons in somatosensory cortex during learning. Nat. Neurosci. 18, 1101–1108. 10.1038/nn.4046 26098757

[B9] De León Reyes N. S. Mederos S. Varela I. Weiss L. A. Perea G. Galazo M. J.. (2019). Transient callosal projections of L4 neurons are eliminated for the acquisition of local connectivity. Nat. Commun. 10:4549. 10.1038/s41467-019-12495-w 31591398 PMC6779895

[B10] Diamond M. E. von Heimendahl M. Knutsen P. M. Kleinfeld D. Ahissar E. (2008). “Where” and “what” in the whisker sensorimotor system. Nat. Rev. Neurosci. 9, 601–612. 10.1038/nrn2411 18641667

[B11] Economo M. N. Clack N. G. Lavis L. D. Gerfen C. R. Svoboda K. Myers E. W.. (2016). A platform for brain-wide imaging and reconstruction of individual neurons. eLife 5:e10566. 10.7554/eLife.10566 26796534 PMC4739768

[B12] Egger R. Narayanan R. T. Guest J. M. Bast A. Udvary D. Messore L. F.. (2020). Cortical output is gated by horizontally projecting neurons in the deep layers. Neuron 105, 122–137.e8. 10.1016/j.neuron.2019.10.011 31784285 PMC6953434

[B13] Feldmeyer D. (2012). Excitatory neuronal connectivity in the barrel cortex. Front. Neuroanat. 6:24. 10.3389/fnana.2012.00024 22798946 PMC3394394

[B14] Feldmeyer D. Lübke J. Sakmann B. (2006). Efficacy and connectivity of intracolumnar pairs of layer 2/3 pyramidal cells in the barrel cortex of juvenile rats. J. Physiol. (Lond.) 575, 583–602. 10.1113/jphysiol.2006.105106 16793907 PMC1819447

[B15] Ferezou I. Haiss F. Gentet L. J. Aronoff R. Weber B. Petersen C. C. H. (2007). Spatiotemporal dynamics of cortical sensorimotor integration in behaving mice. Neuron 56, 907–923. 10.1016/j.neuron.2007.10.007 18054865

[B16] Frick A. Feldmeyer D. Helmstaedter M. Sakmann B. (2008). Monosynaptic connections between pairs of L5A pyramidal neurons in columns of juvenile rat somatosensory cortex. Cereb. Cortex 18, 397–406. 10.1093/cercor/bhm074 17548800

[B17] Frostig R. D. Xiong Y. Chen-Bee C. H. Kvasnák E. Stehberg J. (2008). Large-scale organization of rat sensorimotor cortex based on a motif of large activation spreads. J. Neurosci. 28, 13274–13284. 10.1523/JNEUROSCI.4074-08.2008 19052219 PMC2710304

[B18] Gong H. Xu D. Yuan J. Li X. Guo C. Peng J.. (2016). High-throughput dual-colour precision imaging for brain-wide connectome with cytoarchitectonic landmarks at the cellular level. Nat. Commun. 7:12142. 10.1038/ncomms12142 27374071 PMC4932192

[B19] Grant R. A. Breakell V. Prescott T. J. (2018). Whisker touch sensing guides locomotion in small, quadrupedal mammals. Proc. Biol. Sci. 285:20180592. 10.1098/rspb.2018.0592 29899069 PMC6015872

[B20] Guo C. Peng J. Zhang Y. Li A. Li Y. Yuan J.. (2017). Single-axon level morphological analysis of corticofugal projection neurons in mouse barrel field. Sci. Rep. 7:2846. 10.1038/s41598-017-03000-8 28588276 PMC5460143

[B21] Han Y. Kebschull J. M. Campbell R. A. A. Cowan D. Imhof F. Zador A. M.. (2018). The logic of single-cell projections from visual cortex. Nature 556, 51–56. 10.1038/nature26159 29590093 PMC6585423

[B22] Huang Q. Chen Y. Liu S. Xu C. Cao T. Xu Y.. (2020). Weakly supervised learning of 3D deep network for neuron reconstruction. Front. Neuroanat. 14:38. 10.3389/fnana.2020.00038 32848636 PMC7399060

[B23] Johnson B. A. Frostig R. D. (2016). Long, intrinsic horizontal axons radiating through and beyond rat barrel cortex have spatial distributions similar to horizontal spreads of activity evoked by whisker stimulation. Brain Struct. Funct. 221, 3617–3639. 10.1007/s00429-015-1123-7 26438334 PMC4821813

[B24] Johnson B. A. Frostig R. D. (2018). Long-range, border-crossing, horizontal axon radiations are a common feature of rat neocortical regions that differ in cytoarchitecture. Front. Neuroanat. 12:50. 10.3389/fnana.2018.00050 29977194 PMC6021490

[B25] Judkewitz B. Rizzi M. Kitamura K. Häusser M. (2009). Targeted single-cell electroporation of mammalian neurons *in vivo*. Nat. Protoc. 4, 862–869. 10.1038/nprot.2009.56 19444243

[B26] Koralek K. A. Olavarria J. Killackey H. P. (1990). Areal and laminar organization of corticocortical projections in the rat somatosensory cortex. J. Comp. Neurol. 299, 133–150. 10.1002/cne.902990202 2172324

[B27] Kwon S. E. Yang H. Minamisawa G. O’Connor D. H. (2016). Sensory and decision-related activity propagate in a cortical feedback loop during touch perception. Nat. Neurosci. 19, 1243–1249. 10.1038/nn.4356 27437910 PMC5003632

[B28] Le Merre P. Esmaeili V. Charrière E. Galan K. Salin P.-A. Petersen C. C. H.. (2018). Reward-based learning drives rapid sensory signals in medial prefrontal cortex and dorsal hippocampus necessary for goal-directed behavior. Neuron 97, 83–91.e5. 10.1016/j.neuron.2017.11.031 29249287 PMC5766832

[B29] Lee T. Kim U. (2012). Descending projections from the dysgranular zone of rat primary somatosensory cortex processing deep somatic input. J. Comp. Neurol. 520, 1021–1046. 10.1002/cne.22767 21935942

[B30] Li Q. Shen L. (2020). 3D neuron reconstruction in tangled neuronal image with deep networks. IEEE Trans. Med. Imaging 39, 425–435. 10.1109/TMI.2019.2926568 31295108

[B31] Lübke J. Feldmeyer D. (2007). Excitatory signal flow and connectivity in a cortical column: focus on barrel cortex. Brain Struct. Funct. 212, 3–17. 10.1007/s00429-007-0144-2 17717695

[B32] Magliaro C. Callara A. L. Vanello N. Ahluwalia A. (2019). Gotta trace ‘em all: a mini-review on tools and procedures for segmenting single neurons toward deciphering the structural connectome. Front. Bioeng. Biotechnol. 7:202. 10.3389/fbioe.2019.00202 31555642 PMC6727034

[B33] Mao T. Kusefoglu D. Hooks B. M. Huber D. Petreanu L. Svoboda K. (2011). Long-range neuronal circuits underlying the interaction between sensory and motor cortex. Neuron 72, 111–123. 10.1016/j.neuron.2011.07.029 21982373 PMC5047281

[B34] Matyas F. Sreenivasan V. Marbach F. Wacongne C. Barsy B. Mateo C.. (2010). Motor control by sensory cortex. Science 330, 1240–1243. 10.1126/science.1195797 21109671

[B35] Narayanan R. T. Egger R. Johnson A. S. Mansvelder H. D. Sakmann B. de Kock C. P. J.. (2015). Beyond columnar organization: cell type- and target layer-specific principles of horizontal axon projection patterns in rat vibrissal cortex. Cereb. Cortex 25, 4450–4468. 10.1093/cercor/bhv053 25838038 PMC4816792

[B36] Oberlaender M. Boudewijns Z. S. R. M. Kleele T. Mansvelder H. D. Sakmann B. de Kock C. P. J. (2011). Three-dimensional axon morphologies of individual layer 5 neurons indicate cell type-specific intracortical pathways for whisker motion and touch. Proc. Natl. Acad. Sci. U S A 108, 4188–4193. 10.1073/pnas.1100647108 21368112 PMC3053980

[B640] Oh S. W. Harris J. A. Ng L. Winslow B. Cain M. Mihalas S.. (2014). A mesoscale connectome of the mouse brain. Nature 508, 207–214. 10.1038/nature13186 24695228 PMC5102064

[B37] Pala A. Petersen C. C. H. (2015). *In vivo* measurement of cell-type-specific synaptic connectivity and synaptic transmission in layer 2/3 mouse barrel cortex. Neuron 85, 68–75. 10.1016/j.neuron.2014.11.025 25543458 PMC4305188

[B38] Peng H. Xie P. Liu L. Kuang X. Wang Y. Qu L.. (2021). Morphological diversity of single neurons in molecularly defined cell types. Nature 598, 174–181. 10.1038/s41586-021-03941-1 34616072 PMC8494643

[B39] Petersen C. C. H. (2019). Sensorimotor processing in the rodent barrel cortex. Nat. Rev. Neurosci. 20, 533–546. 10.1038/s41583-019-0200-y 31367018 PMC7116865

[B40] Renier N. Wu Z. Simon D. J. Yang J. Ariel P. Tessier-Lavigne M. (2014). iDISCO: a simple, rapid method to immunolabel large tissue samples for volume imaging. Cell 159, 896–910. 10.1016/j.cell.2014.10.010 25417164

[B41] Rodgers C. C. Nogueira R. Pil B. C. Greeman E. A. Park J. M. Hong Y. K.. (2021). Sensorimotor strategies and neuronal representations for shape discrimination. Neuron 109, 2308–2325.e10. 10.1016/j.neuron.2021.05.019 34133944 PMC8298290

[B42] Rojas-Piloni G. Guest J. M. Egger R. Johnson A. S. Sakmann B. Oberlaender M. (2017). Relationships between structure, *in vivo* function and long-range axonal target of cortical pyramidal tract neurons. Nat. Commun. 8:870. 10.1038/s41467-017-00971-0 29021587 PMC5636900

[B43] Schubert D. Kötter R. Luhmann H. J. Staiger J. F. (2006). Morphology, electrophysiology and functional input connectivity of pyramidal neurons characterizes a genuine layer Va in the primary somatosensory cortex. Cereb. Cortex 16, 223–236. 10.1093/cercor/bhi100 15872153

[B44] Schubert D. Staiger J. F. Cho N. Kötter R. Zilles K. Luhmann H. J. (2001). Layer-specific intracolumnar and transcolumnar functional connectivity of layer V pyramidal cells in rat barrel cortex. J. Neurosci. 21, 3580–3592. 10.1523/JNEUROSCI.21-10-03580.2001 11331387 PMC6762473

[B45] Sippy T. Lapray D. Crochet S. Petersen C. C. H. (2015). Cell-type-specific sensorimotor processing in striatal projection neurons during goal-directed behavior. Neuron 88, 298–305. 10.1016/j.neuron.2015.08.039 26439527 PMC4622932

[B46] Sorensen S. A. Bernard A. Menon V. Royall J. J. Glattfelder K. J. Desta T.. (2015). Correlated gene expression and target specificity demonstrate excitatory projection neuron diversity. Cereb. Cortex 25, 433–449. 10.1093/cercor/bht243 24014670

[B47] Sreenivasan V. Karmakar K. Rijli F. M. Petersen C. C. H. (2015). Parallel pathways from motor and somatosensory cortex for controlling whisker movements in mice. Eur. J. Neurosci. 41, 354–367. 10.1111/ejn.12800 25476605 PMC4359021

[B49] Staiger J. F. Bojak I. Miceli S. Schubert D. (2015). A gradual depth-dependent change in connectivity features of supragranular pyramidal cells in rat barrel cortex. Brain Struct. Funct. 220, 1317–1337. 10.1007/s00429-014-0726-8 24569853 PMC4409644

[B48] Staiger J. F. Petersen C. C. H. (2021). Neuronal circuits in barrel cortex for whisker sensory perception. Physiol. Rev. 101, 353–415. 10.1152/physrev.00019.2019 32816652

[B50] Stehberg J. Dang P. T. Frostig R. D. (2014). Unimodal primary sensory cortices are directly connected by long-range horizontal projections in the rat sensory cortex. Front. Neuroanat. 8:93. 10.3389/fnana.2014.00093 25309339 PMC4174042

[B51] Sumser A. Mease R. A. Sakmann B. Groh A. (2017). Organization and somatotopy of corticothalamic projections from L5B in mouse barrel cortex. Proc. Natl. Acad. Sci. U S A 114, 8853–8858. 10.1073/pnas.1704302114 28774955 PMC5565434

[B52] Susaki E. A. Tainaka K. Perrin D. Yukinaga H. Kuno A. Ueda H. R. (2015). Advanced CUBIC protocols for whole-brain and whole-body clearing and imaging. Nat. Protoc. 10, 1709–1727. 10.1038/nprot.2015.085 26448360

[B53] Vavladeli A. Daigle T. Zeng H. Crochet S. Petersen C. C. H. (2020). Projection-specific activity of layer 2/3 neurons imaged in mouse primary somatosensory barrel cortex during a whisker detection task. Function 1:zqaa008. 10.1093/function/zqaa008 PMC8788860 35330741

[B54] Wang Q. Ding S.-L. Li Y. Royall J. Feng D. Lesnar P.. (2020). The Allen mouse brain common coordinate framework: a 3D reference atlas. Cell 181, 936–953.e20. 10.1016/j.cell.2020.04.007 32386544 PMC8152789

[B55] Wang X. Tucciarone J. Jiang S. Yin F. Wang B.-S. Wang D.. (2019). Genetic single neuron anatomy reveals fine granularity of cortical axo-axonic cells. Cell Rep. 26, 3145–3159.e5. 10.1016/j.celrep.2019.02.040 30865900 PMC7863572

[B56] Welker E. Hoogland P. V. Van der Loos H. (1988). Organization of feedback and feedforward projections of the barrel cortex: a PHA-L study in the mouse. Exp. Brain Res. 73, 411–435. 10.1007/BF00248234 3215316

[B57] White E. L. DeAmicis R. A. (1977). Afferent and efferent projections of the region in mouse SmL cortex which contains the posteromedial barrel subfield. J. Comp. Neurol. 175, 455–482. 10.1002/cne.901750405 915034

[B58] Winnubst J. Bas E. Ferreira T. A. Wu Z. Economo M. N. Edson P.. (2019). Reconstruction of 1,000 projection neurons reveals new cell types and organization of long-range connectivity in the mouse brain. Cell 179, 268–281.e13. 10.1016/j.cell.2019.07.042 31495573 PMC6754285

[B59] Woolsey T. A. Van der Loos H. (1970). The structural organization of layer IV in the somatosensory region (SI) of mouse cerebral cortex. The description of a cortical field composed of discrete cytoarchitectonic units. Brain Res. 17, 205–242. 10.1016/0006-8993(70)90079-x 4904874

[B61] Yamashita T. Pala A. Pedrido L. Kremer Y. Welker E. Petersen C. C. H. (2013). Membrane potential dynamics of neocortical projection neurons driving target-specific signals. Neuron 80, 1477–1490. 10.1016/j.neuron.2013.10.059 24360548

[B60] Yamashita T. Petersen C. C. H. (2016). Target-specific membrane potential dynamics of neocortical projection neurons during goal-directed behavior. eLife 5:e15798. 10.7554/eLife.15798 27328320 PMC4915810

[B62] Yamashita T. Vavladeli A. Pala A. Galan K. Crochet S. Petersen S. S. A.. (2018). Diverse long-range axonal projections of excitatory layer 2/3 neurons in mouse barrel cortex. Front. Neuroanat. 12:33. 10.3389/fnana.2018.00033 29765308 PMC5938399

[B63] Zakiewicz I. Bjaalie J. Leergaard T. (2014). Brain-wide map of efferent projections from rat barrel cortex. Front. Neuroinform. 8:5. 10.3389/fninf.2014.00005 24550819 PMC3914153

[B64] Zhou Z. Kuo H.-C. Peng H. Long F. (2018). DeepNeuron: an open deep learning toolbox for neuron tracing. Brain Inform. 5:3. 10.1186/s40708-018-0081-2 29876679 PMC5990497

[B65] Zingg B. Hintiryan H. Gou L. Song M. Y. Bay M. Bienkowski M. S.. (2014). Neural networks of the mouse neocortex. Cell 156, 1096–1111. 10.1016/j.cell.2014.02.023 24581503 PMC4169118

