# Supplementary material for: Axonal and Dendritic Morphology of Excitatory Neurons in Layer 2/3 Mouse Barrel Cortex Imaged Through Whole-Brain Two-Photon Tomography and Registered to a Digital Brain Atlas
Source: Front Neuroanat. 2022 Jan 25;15:791015. doi: 10.3389/fnana.2021.791015 (PMC8821665; doi:10.3389/fnana.2021.791015)
Supplement: Supplementary file 1 [file Data_Sheet_1.PDF]

Supplementary Information

Axonal and dendritic morphology of excitatory neurons in layer 2/3 mouse barrel cortex imaged through whole-brain two-photon tomography and registered to a digital brain atlas

Yanqi Liu, Georgios Foustoukos, Sylvain Crochet and Carl Petersen

Supplementary Information consists of:

- Supplementary Figure 1
- Supplementary Figure 2
- Supplementary Figure 3
- Supplementary Figure 4
- Supplementary Figure 5
- Supplementary Figure 6
- Supplementary Figure 7
- Supplementary Table 1

Supplementary Figure 1

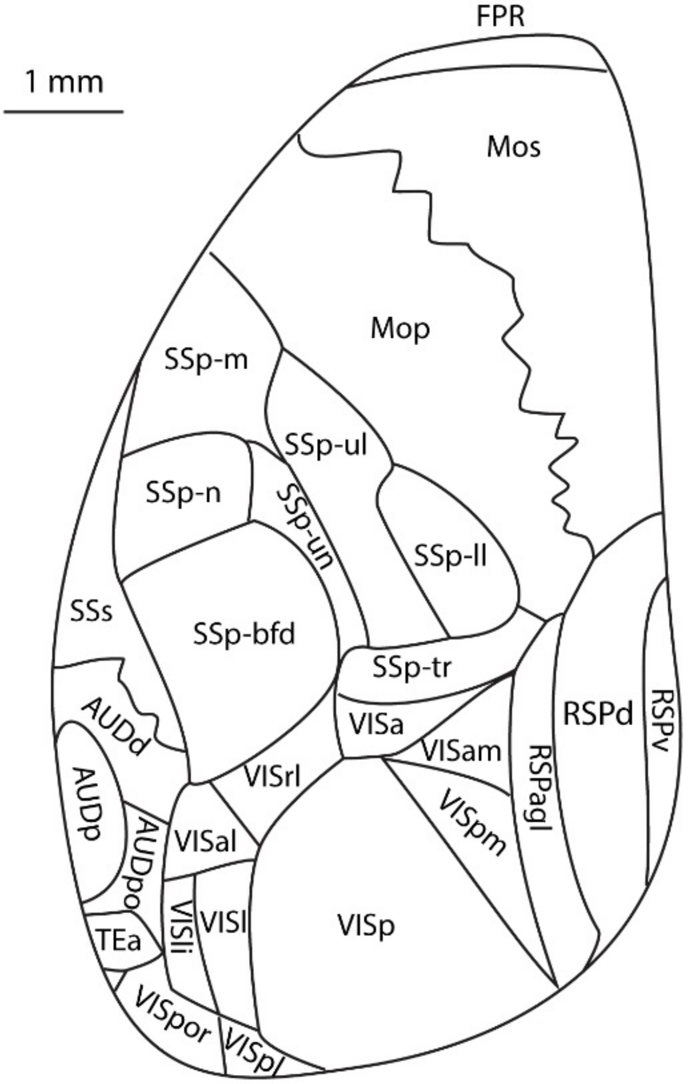

Supplementary Figure 1. Anatomical locations of different cortical areas in a horizontal view of the mouse dorsal cortex. For definitions of area acronyms, please see Supplementary Table 1.

Supplementary Figure 2

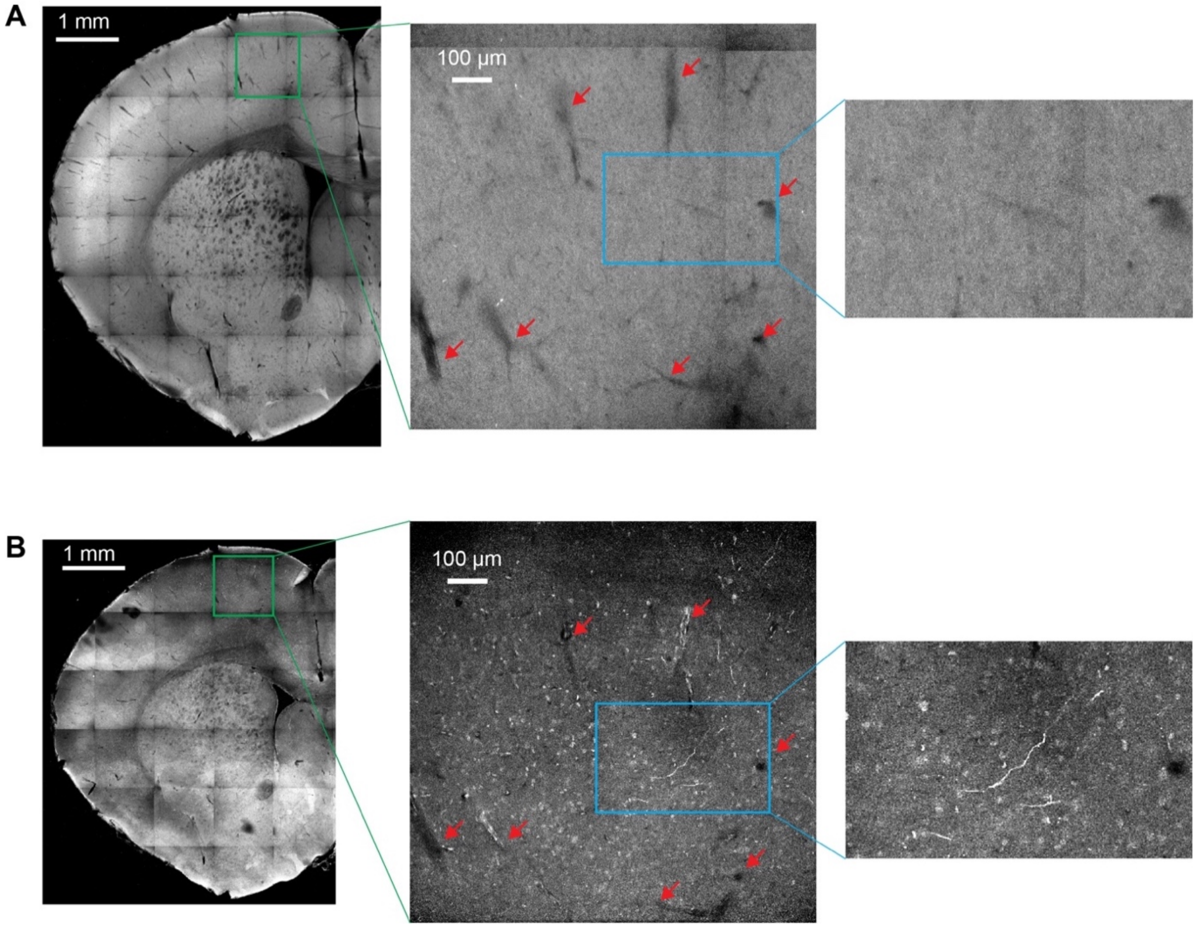

Supplementary Figure 2. Example section demonstrating that antibody staining reveals axonal structures not found in the two-photon tomography. **(A)** *Left*, overview image acquired with two-photon tomography with a region of interest (ROI) indicated with a green frame. *Middle*, zoomed in view of the green ROI with arrows indicating blood vessel landmarks and a smaller ROI indicated with a blue frame. *Right*, zoomed in view of the blue ROI. **(B)** *Left*, overview of the same brain slice as (A) after signal amplification with anti-GFP antibody, mounted on a glass coverslip and imaged with the two-photon tomographic microscope. A region of interest (ROI) is indicated with a green frame. *Middle*, zoomed in view of the same green ROI as in (A), identified by blood vessel patterns (arrows). Both images are averaged images of 50 µm z-stacks. Note that the brain slice underwent shrinking and distortions during the immunohistochemical staining and mounting, thus landmarks do not match perfectly. A smaller ROI, similar to (A) is indicated with a blue frame. *Right*, zoomed in of the blue ROI. Note the presence of labelled axon segments in B that are not visible in A.

Supplementary Figure 3

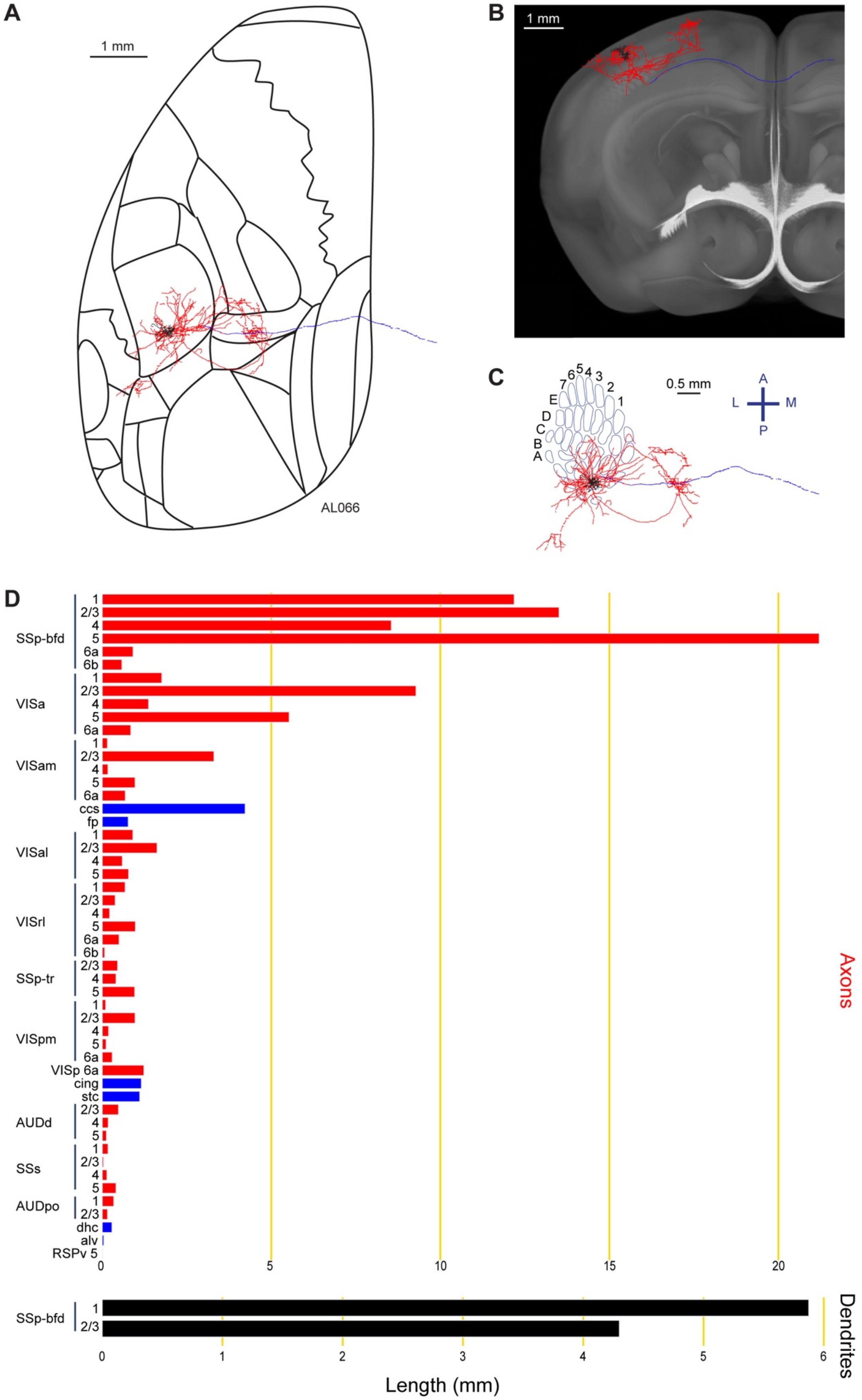

Supplementary Figure 3. Reconstruction and quantification of example neuron AL066 with projections to visual areas. **(A)** Maximum projection of reconstructed axon and dendrites in horizontal view, aligned to the Allen Mouse CCFv3 to indicate boundaries between cortical regions. **(B)** Maximum projection of axon and dendrites in coronal view. **(C)** Maximum projection of axons and dendrites in tangential view (rotated 30 degrees) over the barrel field. The cell body was located in the B1 barrel column. **(D)** Quantification of axonal (*top*) and dendritic (*bottom*) length in respective brain regions identified by the Allen Mouse CCFv3. Dendrites are shown in black; axons in neocortical grey matter are shown in red; and axons in white matter are shown in blue.

Supplementary Figure 4

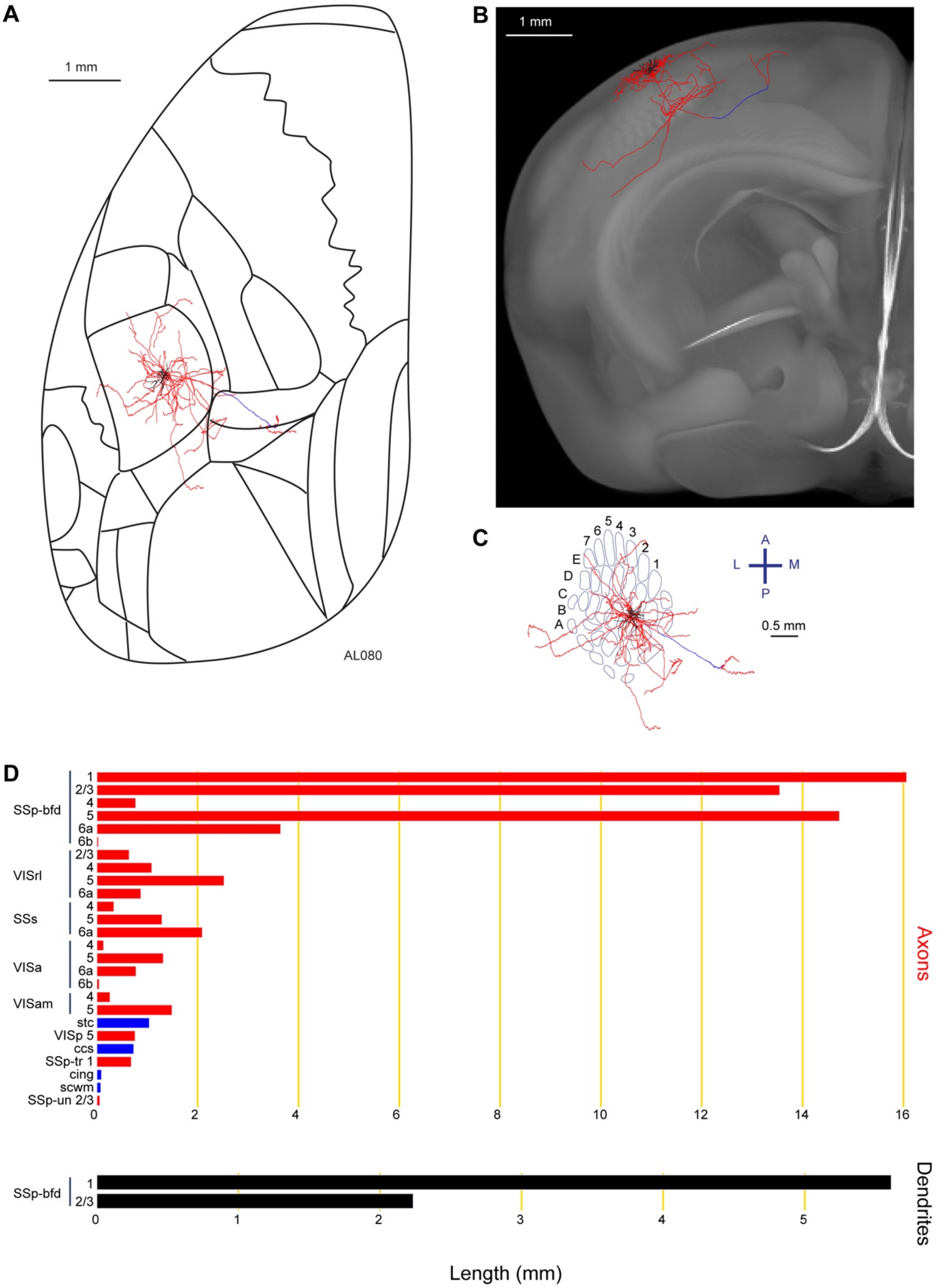

Supplementary Figure 4. Reconstruction and quantification of example neuron AL080 with projections to visual areas and the secondary somatosensory cortex. **(A)** Maximum projection of axon and dendrites in horizontal view. **(B)** Maximum projection of axon and dendrites in coronal view. **(C)** Maximum projection of axon and dendrites in tangential view (rotated 30 degrees) over the barrel field. The soma of the neuron was located in the septa between the C2 and D2 barrel columns. **(D)** Quantification of axonal (top) and dendritic (bottom) length in respective brain regions identified by the Allen Mouse CCFv3. Dendrites are shown in black; axons in neocortical grey matter are shown in red; and axons in white matter are shown in blue.

Supplementary Figure 5

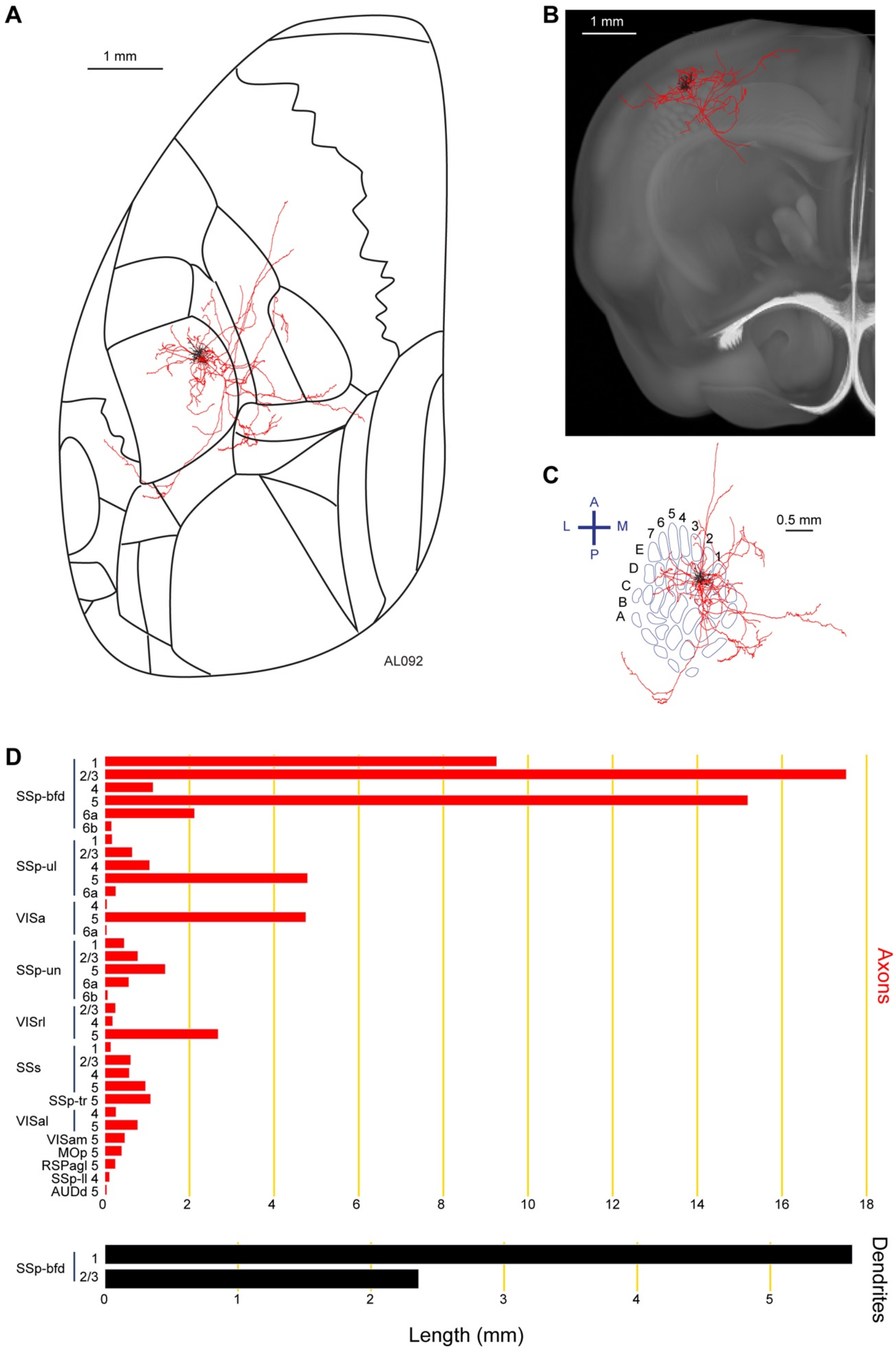

Supplementary Figure 5. Reconstruction and quantification of example neuron AL092 with projections to the primary somatosensory area upper limb area. **(A)** Maximum projection of axon and dendrites in horizontal view. **(B)** Maximum projection of axon and dendrites in coronal view. **(C)** Maximum projection of axon and dendrites in tangential view (rotated 30 degrees) over the barrel field. The cell body was located in the D3 barrel column. **(D)** Quantification of axonal (top) and dendritic (bottom) length in respective brain regions identified by the Allen Mouse CCFv3. Dendrites are shown in black; and axon in neocortical grey matter is shown in red.

Supplementary Figure 6

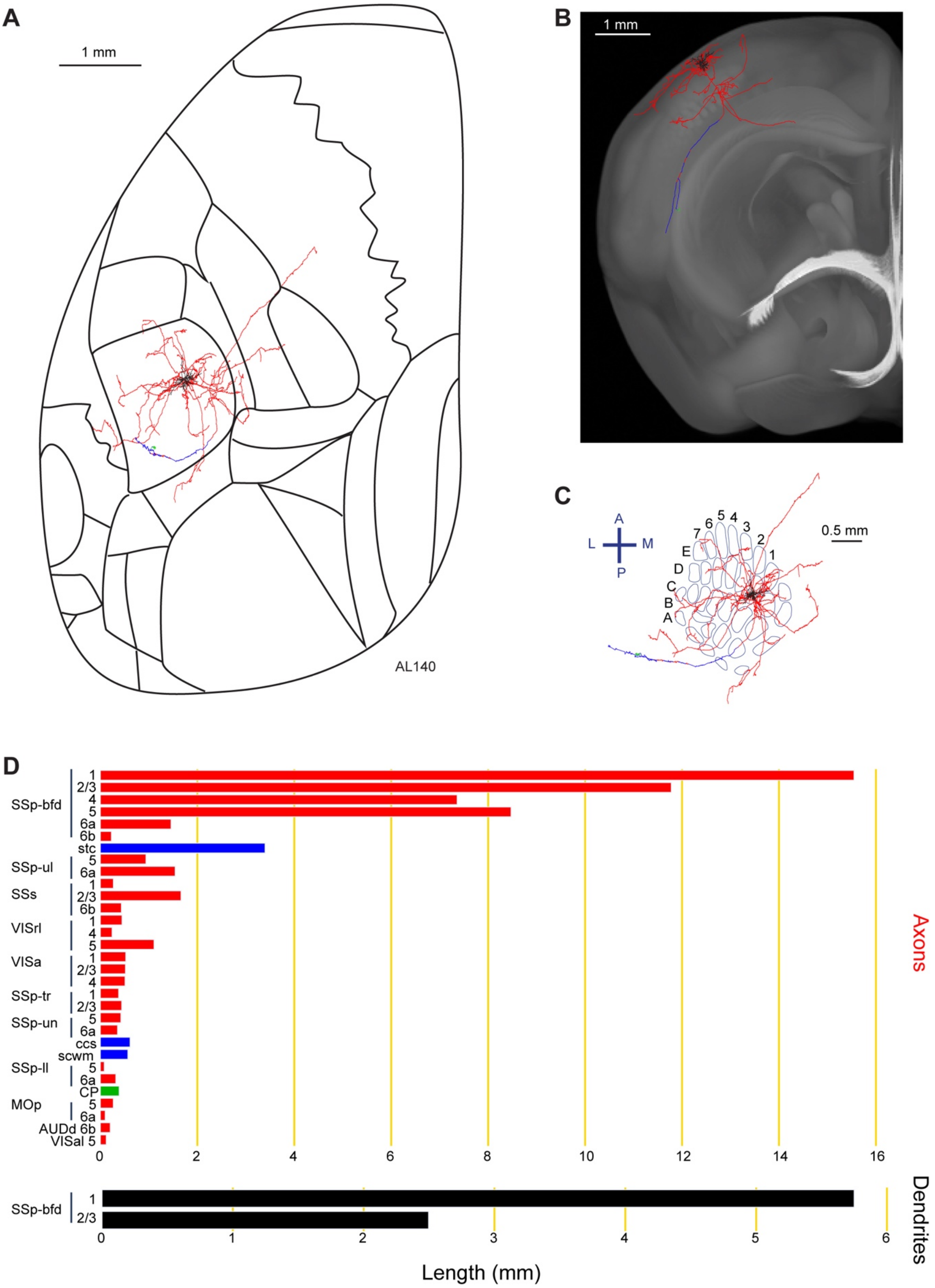

Supplementary Figure 6. Reconstruction and quantification of example neuron AL140 with projections to the primary somatosensory upper limb area, the secondary somatosensory area and visual areas. **(A)** Maximum projection of axon and dendrites in horizontal view. **(B)** Maximum projection of axon and dendrites in coronal view. **(C)** Maximum projection of axon and dendrites in tangential view (rotated 30 degrees) over the barrel field. The cell body was in the D2 barrel column. **(D)** Quantification of axonal (top) and dendritic (bottom) length in respective brain regions identified by the Allen Mouse CCFv3. Dendrites are shown in black; axon in neocortical grey matter is shown in red; axon in striatum is shown in green; and axon in white matter is shown in blue.

Supplementary Figure 7

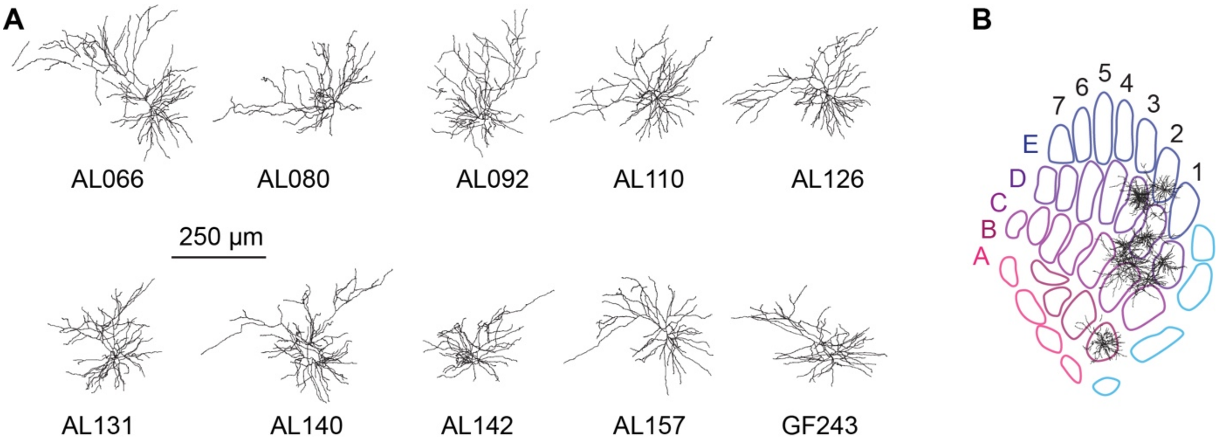

Supplementary Figure 7. Dendritic morphologies. **(A)** Reconstruction of dendrites of all neurons in coronal representations (n = 10). **(B)** Location of dendrites superimposed on top of the mouse barrel field with arcs (1-7) and rows (A-E) labelled.

Supplementary Table 1

SSp-bfd	Primary somatosensory area, barrel field
SSp-bfd1	Primary somatosensory area, barrel field, layer 1
SSp-bfd2/3	Primary somatosensory area, barrel field, layer 2/3
SSp-bfd5	Primary somatosensory area, barrel field, layer 5
SSp-bfd4	Primary somatosensory area, barrel field, layer 4
SSp-bfd6a	Primary somatosensory area, barrel field, layer 6a
SSp-bfd6b	Primary somatosensory area, barrel field, layer 6b
SSp-ul	Primary somatosensory area, upper limb
SSp-ul1	Primary somatosensory area, upper limb, layer 1
SSp-ul2/3	Primary somatosensory area, upper limb, layer 2/3
SSp-ul4	Primary somatosensory area, upper limb, layer 4
SSp-ul5	Primary somatosensory area, upper limb, layer 5
SSp-ul6a	Primary somatosensory area, upper limb, layer 6a
SSp-ul6b	Primary somatosensory area, upper limb, layer 6b
SSs	Supplemental somatosensory area
SSs1	Supplemental somatosensory area, layer 1
SSs2/3	Supplemental somatosensory area, layer 2/3
SSs4	Supplemental somatosensory area, layer 4
SSs5	Supplemental somatosensory area, layer 5
SSs6a	Supplemental somatosensory area, layer 6a
SSs6b	Supplemental somatosensory area, layer 6b
SSp-un	Primary somatosensory area, unassigned
SSp-un1	Primary somatosensory area, unassigned, layer 1
SSp-un2/3	Primary somatosensory area, unassigned, layer 2/3
SSp-un4	Primary somatosensory area, unassigned, layer 4
SSp-un5	Primary somatosensory area, unassigned, layer 5
SSp-un6a	Primary somatosensory area, unassigned, layer 6a
SSp-un6b	Primary somatosensory area, unassigned, layer 6b
SSp-tr	Primary somatosensory area, trunk
SSp-tr1	Primary somatosensory area, trunk, layer 1
SSp-tr2/3	Primary somatosensory area, trunk, layer 2/3
SSp-tr4	Primary somatosensory area, trunk, layer 4
SSp-tr5	Primary somatosensory area, trunk, layer 5
SSp-tr6a	Primary somatosensory area, trunk, layer 6a
SSp-tr6b	Primary somatosensory area, trunk, layer 6b
SSp-ll	Primary somatosensory area, lower limb
SSp-ll2/3	Primary somatosensory area, lower limb, layer 2/3
SSp-ll4	Primary somatosensory area, lower limb, layer 4
SSp-ll5	Primary somatosensory area, lower limb, layer 5
SSp-ll6a	Primary somatosensory area, lower limb, layer 6a

SSp-n	Primary somatosensory area, nose
SSp-n6a	Primary somatosensory area, nose, layer 6a
SSp-n6b	Primary somatosensory area, nose, layer 6b
VISp	Primary visual area
VISp5	Primary visual area, layer 5
VISp6a	Primary visual area, layer 6a
VISpm	posteromedial visual area
VISpm1	posteromedial visual area, layer 1
VISpm2/3	posteromedial visual area, layer 2/3
VISpm4	posteromedial visual area, layer 4
VISpm6a	posteromedial visual area, layer 6a
VISrl	Rostrolateral visual area
VISrl1	Rostrolateral area, layer 1
VISrl2/3	Rostrolateral area, layer 2/3
VISrl4	Rostrolateral area, layer 4
VISrl5	Rostrolateral area, layer 5
VISrl6a	Rostrolateral area, layer 6a
VISrl6b	Rostrolateral area, layer 6b
VISal	Anterolateral visual area
VISal1	Anterolateral visual area, layer 1
VISal2/3	Anterolateral visual area, layer 2/3
VISal4	Anterolateral visual area, layer 4
VISal5	Anterolateral visual area, layer 5
VISam	Anteromedial visual area
VISam1	Anteromedial visual area, layer 1
VISam2/3	Anteromedial visual area, layer 2/3
VISam4	Anteromedial visual area, layer 4
VISam5	Anteromedial visual area, layer 5
VISam6a	Anteromedial visual area, layer 6a
VISa	Anterior area
VISa1	Anterior area, layer 1
VISa2/3	Anterior area, layer 2/3
VISa4	Anterior area, layer 4
VISa5	Anterior area, layer 5
VISa6b	Anterior area, layer 6b
MOp	Primary motor area
MOp5	Primary motor area, Layer 5
MOp6a	Primary motor area, Layer 6a
MOp6b	Primary motor area, Layer 6b
MOs	Secondary motor area
MOs6a	Secondary motor area, layer 6a
MOs6b	Secondary motor area, layer 6b
AUDd	Dorsal auditory area

AUDd2/3	Dorsal auditory area, layer 2/3
AUDd4	Dorsal auditory area, layer 4
AUDd5	Dorsal auditory area, layer 5
AUDd6b	Dorsal auditory area, layer 6b
AUDpo	Posterior auditory area
AUDpo1	Posterior auditory area, layer 1
AUDpo2/3	Posterior auditory area, layer 2/3
VISC	Visceral area
VISC6a	Visceral area, layer 6a
VISC6b	Visceral area, layer 6b
hc	hippocampal commissures
dhc	dorsal hippocampal commissure
RSPagl	Retrosplenial area, lateral agranular part
RSPagl5	Retrosplenial area, lateral agranular part, layer 5
cc	corpus callosum
ccb	corpus callosum, body
ccs	corpus callosum, splenium
fp	corpus callosum, posterior forceps
fa	corpus callosum, anterior forceps
fxs	fornix system
RSPv	Retrosplenial area, ventral part
RSPv5	Retrosplenial area, ventral part, layer 5
STRd	Striatum dorsal region
CP	Caudoputamen
cing	cingulum bundle
ec	external capsule
alv	alveus
hc	hippocampal commissures
dhc	dorsal hippocampal commissure
RSPagl	Retrosplenial area, lateral agranular part
St	stria terminalis
stc	commissural branch of stria terminalis
scwm	supra-callosal cerebral white matter
fiber tracts	fiber tracts
mfbc	cerebrum related

Supplementary Table 1. List of brain areas and their abbreviations in the Allen Common Coordinate Framework (CCFv3) appearing in the figures of our study.
